# Combined targeting of pathways regulating synaptic formation and autophagy attenuates Alzheimer’s disease pathology in mice

**DOI:** 10.3389/fphar.2022.913971

**Published:** 2022-08-16

**Authors:** Christiana Bjorkli, Mary Hemler, Joshua B. Julian, Axel Sandvig, Ioanna Sandvig

**Affiliations:** ^1^ Department of Neuromedicine and Movement Science, Faculty of Medicine and Health Sciences, Norwegian University of Science and Technology, Trondheim, Norway; ^2^ Department of Neurology, St. Olav’s Hospital, Trondheim, Norway; ^3^ Princeton Neuroscience Institute, Princeton University, Princeton, NJ, United States; ^4^ Department of Clinical Neurosciences, Division of Neuro Head and Neck, Umeå University Hospital, Umeå, Sweden; ^5^ Department of Community Medicine and Rehabilitation, Umeå University, Umeå, Sweden

**Keywords:** microdialysis, repurposed drugs, Wnt signalling, mTor pathway, amyloid plaques, neurofibrillary tangles

## Abstract

All drug trials completed to date have fallen short of meeting the clinical endpoint of significantly slowing cognitive decline in Alzheimer’s disease (AD) patients. In this study, we repurposed two FDA-approved drugs, Fasudil and Lonafarnib, targeting synaptic formation (i.e., Wnt signaling) and cellular clearance (i.e., autophagic) pathways respectively, to test their therapeutic potential for attenuating AD-related pathology. We characterized our 3xTg AD mouse colony to select timepoints for separate and combinatorial treatment of both drugs while collecting cerebrospinal fluid (CSF) using an optimized microdialysis method. We found that treatment with Fasudil reduced Aβ at early and later stages of AD, whereas administration of Lonafarnib had no effect on Aβ, but did reduce tau, at early stages of the disease. Induction of autophagy led to increased size of amyloid plaques when administered at late phases of the disease. We show that combinatorial treatment with both drugs was effective at reducing intraneuronal Aβ and led to improved cognitive performance in mice. These findings lend support to regulating Wnt and autophagic pathways in order to attenuate AD-related pathology.

## Introduction

Alzheimer’s disease (AD) is the leading cause of dementia, and symptoms include progressive neurodegeneration, followed by impairments in memory, cognitive and visuospatial function (De Strooper and Karran, 2016). Neuropathology in AD patients is characterized by extracellular deposits of aggregated amyloid-β (Aβ; i.e., amyloid plaques), and neurofibrillary tangles (NFTs) consisting of hyperphosphorylated tau, and eventually abiotrophic neuronal cell death starting in superficial layers of lateral entorhinal cortex (LEC) (Braak and Braak, 1991; Thal et al., 2002; De Strooper and Karran, 2016). Both amyloid plaques and NFTs are believed to disrupt the healthy function of synapses and neurons (Mufson et al., 2016), as well as to impair cellular clearance along autophagic pathways (Baixauli et al., 2014; Liu and Li, 2019). Researchers and clinicians are currently utilizing cerebrospinal fluid (CSF) levels of Aβ and tau to predict the development and rate of the disease (Snider et al., 2009; Buchhave et al., 2012; Mufson et al., 2016; Bjorkli et al., 2020).

Over the years there have been multiple therapeutic approaches aimed at lowering Aβ and tau levels. Promising avenues for AD therapeutics include drugs that attenuate synaptic loss and therapies that take advantage of the autophagic system to remove intraneuronal protein aggerates. Both drug targets represent structural and biochemical correlates of cognitive decline in AD. For instance, Aβ-driven synaptic loss has been found to be dependent on the activation of a branch of Wnt^1^ signaling known as the Wnt-planar cell polarity (Wnt-PCP) pathway (Killick et al., 2014) (Supplementary Figure S1A). Drugs that inhibit Rho-associated protein kinase (ROCK) along the Wnt-PCP pathway have been shown to attenuate AD-related pathology, and these include pitavastatin (Hamano et al., 2020), FSD-C10 (Gu et al., 2018), Y-27632 (Herskowitz et al., 2013), and Fasudil (Tang and Liou, 2007; Herskowitz et al., 2013; Elliott et al., 2018; Sellers et al., 2018), among others. Aβ has been shown to activate Wnt-PCP signaling through its ability to induce Dickkopf-1 (Dkk1), which then blocks the binding interaction between lipoprotein receptor-related protein 6 (LRP6) and frizzled, leading to activation of Wnt-PCP signaling in favor of the canonical Wnt pathway (Bafico et al., 2001; Sellers et al., 2018) (Supplementary Figure S1A). Along the same arm of Wnt-PCP signaling, binding of a Wnt receptor to frizzled causes disheveled to inhibit the activity of glycogen synthase kinase-3β (GSK-3β) (Moon et al., 2004), and this serine/threonine kinase contributes to the hyperphosphorylation of tau proteins (Hanger et al., 1992). Fasudil is clinically approved to treat cerebral consequences of subarachnoid haemorrhage, and as a ROCK inhibitor (Supplementary Figure S1A) it shows promise in halting the deleterious downstream effects of Aβ and tau pathology. Fasudil inhibits the arm of the Wnt-PCP pathway that promotes the retraction of dendritic spines and synapses through the disheveled associated activator of morphogenesis 1 (Daam1)/Ras homolog family member A (RhoA)/ROCK pathway (Ethell and Pasquale, 2005; Schratt, 2009).

Similarly, it has been shown that autophagic activators can lower the levels of misfolded and aggregated proteins, prevent the spread of tau, and reduce neuronal loss in experimental models (Schaeffer et al., 2012; Siman et al., 2015; Hernandez et al., 2019). Drugs that induce autophagy have been shown to attenuate AD-related pathology, and includes rapamycin (Majumder et al., 2011; Zhang et al., 2017), carbamazepine (Zhang et al., 2017), lithium (Forlenza et al., 2012), memantine (Song et al., 2015), nicotinamide (Liu et al., 2013), resveratrol (Porquet et al., 2014), and Lonafarnib (Hernandez et al., 2019), as well as others. For instance, activation of mammalian target of rapamycin (mTOR) results in activation of downstream components (i.e., 4EBP1 and p70S6K1) (Supplementary Figure S1B **)** which both initiate cascades resulting in tau hyperphosphorylation and eventual NFTs. mTOR activation also leads to the accumulation of amyloid plaques by inhibiting autophagy, while accumulated Aβ further induces tau phosphorylation and mTOR activation (Mueed et al., 2019). Lonafarnib is a clinically approved cancer drug, and works as a farnesyltransferase^2^ inhibitor, which acts as an autophagic inducer by inhibiting mTOR and prevents the accumulation of aggregated proteins and reduces cognitive decline (Pan and Yeung, 2005) (Supplementary Figure S1B). The mechanisms of action involves Ras homologue enriched in brain (Rheb) and the phosphatidylinositide 3-kinase (PI3K)/protein kinase B (Akt)/mTOR pathway. Rheb acts downstream of tuberous sclerosis complex 1 (TSC1)/TSC2 and upstream of mTOR to regulate cell growth (Inoki et al., 2003) and activates S6 kinase 1 during amino acid deprivation via mTOR (Avruch et al., 2006).

To date, drugs have fallen short of meeting the clinical endpoint of significantly slowing cognitive decline in AD patients (Gauthier et al., 2016), possibly due to off-target effects since most drugs are unable to cross the blood-brain barrier (BBB). One way of systemically delivering drugs into the brain is by *in vivo* microdialysis (Deguchi, 2002). Here we have used the most complete transgenic mouse model for AD to date, the 3xTg AD mouse, which has been shown by us and others to develop age-dependent amyloid plaque and NFT deposition (Oddo et al., 2003; Javonillo et al., 2022). We used an optimized protocol for intraventricular microdialysis (Bjorkli et al., 2021) to systemically target two promising therapeutic avenues, the Wnt and autophagic pathways, to attenuate Aβ and tau levels respectively, at the molecular and functional level. Many drugs that target these two pathways have displayed adverse side-effects in experimental models and patients, and we therefore opted to repurpose drugs that were already approved for clinical use. Fasudil and Lonafarnib were also chosen because they target independent pathways that regulate and/or is affected by both Aβ and tau pathology. Here we demonstrate that novel combinatorial treatment with both drugs was effective at reducing intraneuronal Aβ and led to improved context-dependent spatial memory in mice.

## Materials and methods

### Animals

Thirty 3xTg AD mice (MMRRC Strain #034830-JAX; RRID:MMRRC_034830-MU) (Oddo et al., 2003; Billings et al., 2005) and two control B6129 mice (Strain #:101045; RRID: IMSR_JAX:101045) were included in these experiments. 3xTg AD mice contain three mutations associated with familial Alzheimer’s disease (*APP*_*Swe*_, *MAPT*_*P301L*_, and *PSEN1*_*M146V*_). The donating investigators of the 3xTg AD mouse model have previously communicated to Jackson Laboratories that male transgenic mice may not exhibit all phenotypic traits of AD (Billings et al., 2005). Therefore, only female mice were included in these experiments. See Supplementary Table S1 for key resources used, and Supplementary Table S2 for an overview of the sample size for each experimental condition.

To validate whether our mouse model replicated AD neuropathology as observed in patients, and to assess the possibility of genetic drift in our own colony (Zeldovich, 2017), we characterized the 3xTg AD mouse model. Our findings suggest that genetic drift with phenotypic effects (Masel, 2011) occurred in our mouse colony, but we also confirm that this mouse presents as a valid model for studying AD-related neuropathology.

All housing and breeding of animals was approved by the Norwegian Animal Research Authority and is in accordance with the Norwegian Animal Welfare Act §§ 1-28, the Norwegian Regulations of Animal Research §§ 1-26, and the European Convention for the Protection of Vertebrate Animals used for Experimental and Other Scientific Purposes (FOTS ID 21061). The animals were kept on a 12 h light/dark cycle under standard laboratory conditions (19–22°C, 50%–60% humidity), and had free access to food and water.

### Microdialysis guide implantation surgeries

Surgeries were based on previously established protocols (Bjorkli, 2021; Bjorkli et al., 2021), but in brief, implantation surgery was performed to insert microdialysis guide cannulas (CMA 7; CMA Microdialysis AB, Kista, Sweden) into the lateral ventricle of mice. Mice were anesthetized with isoflurane gas (4% induction and 1.5–3% maintenance; IsoFlo vet., Abbott Laboratories, Chicago, IL, United States) prior to being fixed in a stereotaxic frame (Kopf Instruments; Chicago, IL, United States). Prior to making any incisions, Marcain (0.03–0.18 mg/kg; Aspen Pharma, Ballerup, Denmark) was injected subcutaneously into the scalp and Metacam (5 mg/kg; Boehringer Ingelheim Vetmedia, Copenhagen, Denmark) and Temgesic (0.05–0.1 mg/kg; Invidor United Kingdom, Slough, Great Britain) were administered subcutaneously for intraoperative pain relief. Equal heights of bregma and lambda were measured to ensure that the skull was level for each animal (with ± 0.1 mm tolerance), as well as two points equally distant from the midline. After leveling the skull, the stereotaxic coordinates were derived to target the lateral ventricle (A/P: −0.1 mm, M/L: +1.2 mm, D/V: −2.75 mm; see Supplementary Figure S2 for histological verification of probe placement). The microdialysis guide cannula was attached to the stereotaxic frame using a guide clip and connection rod for the clip (CMA Microdialysis AB, Kista, Sweden). The skull was drilled through at these coordinates and the guide cannula was slowly lowered into the drilled hole. The guide cannula was attached to the skull with super glue and dental cement (Dentalon Plus; Cliniclands AB, Trelleborg, Sweden). Post-surgery, Metacam and Temgesic were administered within 24 h. The guide cannula was implanted into the right hemisphere of all animals, as we did not observe any lateralization of pathology in the brains of 3xTg AD mice (Supplementary Figure S3).

### Stereotactic viral injections of P301L tau

Mice were treated identically to microdialysis guide implantation surgeries, up until deriving stereotaxic coordinates. To target LEC layer II, a craniotomy was made at 0.5 mm anterior to lambda and ∼4 mm lateral (dependent on animal weight) to the midline. A Hamilton microsyringe (Neuros 32-gauge syringe, 5 μl, Hamilton company, Nevada, United States) was lowered vertically into the brain to a depth ∼3.6 mm (dependent on animal weight) from the surface, and 300–1,500 nl of viruses was injected using a microinjector (Nanoliter 2010, World Precision Instruments Inc., United States). We injected the adeno-associated virus (AAV)8 GFP-2a-P301Ltau (with the chicken beta actin [CBA] promoter; hereafter referred to as AAV-tau), generated by Dr. Nair at the Viral Vector Core Facility, at the Kavli Institute for Systems Neuroscience, in Trondheim, Norway. More information regarding AAV-tau can be found in this paper (Wegmann et al., 2019), but in brief, the short 2A peptide cleaves GFP and human tau during translation at the ribosome (Szymczak et al., 2004). This results in neurons transduced with the virus being able to produce GFP and human tau as individual proteins (GFP+/MC1+; donor neurons). Conversely, neurons that receive human tau from cross-neuronal spread have human tau, but no GFP (GFP-/MC1+; recipient neurons). The microsyringe was kept in place for 5 min prior and after the injection, to minimize potential upward leakage of the viral solution. Metacam was given within 24 h post-surgery. Animals were implanted with microdialysis guide cannulas 2 months following injections.

### Push-pull microdialysis apparatus and sampling

Push-pull microdialysis was conducted as previously described (Bjorkli et al., 2021), but in brief a refrigerated fraction collector (CMA 470) was set to 6°C for the storage of collected CSF in 300 µl low-retention polypropylene plastic vials (Harvard Apparatus, Cambridge, MA, United States). Fluorinated ethylene propylene (FEP) peristaltic tubing (CMA Microdialysis AB, Kista, Sweden) was placed inside each plastic vial for collection and connected to the cassette of the peristaltic roller pump (Reglo ICC Digital). This peristaltic FEP tubing was connected to the outlet side of microdialysis probes (β-irrigated 2 mDa microdialysis probe; CMA 7; CMA Microdialysis AB, Kista, Sweden) with a polyethersulfone 2 mm membrane with tubing adapters bathed in 75% ethanol. FEP tubing (CMA Microdialysis AB, Kista, Sweden) was connected to each microsyringe. The FEP tubing was then connected to the inlet part of the microdialysis probes. Transparent cages were prepared with 1.5 cm of bedding, filled water bottles, and treats. Saline or drugs were loaded inside a gastight microsyringe (CMA Microdialysis AB, Kista, Sweden), which was placed into a syringe pump (CMA 4004). The “dead volume” of the FEP outlet tubing (1.2 ml/100 mm) was calculated. 100 cm of FEP outlet tubing was used, and therefore the first 12 ml sampled from each animal were discarded. Prior to inserting the microdialysis probes into the guide cannula, the probe was conditioned in 75% ethanol for better recovery of analytes. At the conclusion of microdialyte sampling, the vials of 60 µl CSF were centrifuged and kept at −80°C until the samples were analyzed with multiplex ELISA.

### Intraventricular drug infusions

Previously, researchers have administered a dosage of 10 mg/kg of Fasudil into the lateral ventricle (Sellers et al., 2018) to attenuate Aβ levels, and 80 mg/kg of Lonafarnib orally to attenuate tau levels in mice (Hernandez et al., 2019). Both drugs have previously been delivered using DMSO, which can damage the BBB and mitochondria as well as cause apoptosis (Yuan et al., 2014). Since we had a less effective delivery vehicle than DMSO (Blevins et al., 2002), we conducted pilot experiments to determine effective titers of Fasudil and Lonafarnib, as well as to determine the most effective duration of infusions. Previous research has shown that ∼98% of all small molecules are not transported across the BBB (Pardridge, 2005), whilst other research has shown poor drug transport from CSF to the brain (Yan et al., 1994). Taking drug transport across the BBB, and from CSF into the brain parenchyma into consideration, dosages of both 25, 50 and 80 mg/kg were administered in initial pilot experiments.

In these experiments, a final concentration of 50 mg/kg of Fasudil (10 mM; Selleck Chemicals, Houston, TX, United States) was infused for 14 days in mice and stored at –80°C, whilst a final concentration of 80 mg/kg of Lonafarnib (5 mM; Cayman Chemical, Ann Arbor, MI, United States) was infused for 10 days and stored −20°C between infusions (see Supplementary Table S1 for key resources used). These drug concentrations resulted in no observable side-effects in mice. The same dosages were used during combinatorial infusions of Fasudil and Lonafarnib (administered for a duration of 7 days) in mice (*n* = 4) as the drugs target independent intracellular pathways. All dosages in ml were calculated using; dosage (mg)/concentration (mg/ml) = dose x ml) and were infused at a volume of 60 μl at a rate of 1 μl/min using saline as a control vehicle.

To assess the efficacy of oral versus intraventricular drug administration, we mixed 0.6 ml of 50 mg/kg Fasudil and 80 mg/kg Lonafarnib in baby porridge (Nestlé S.A., Vevey, Switzerland) in 3xTg AD mice (*n* = 3). Intraventricular drug infusions were more effective in reducing intraneuronal Aβ accumulation in the dorsal subiculum (dSub) compared to oral administration of the drugs (t_65_ = 2.54, *p* = 0.0136, unpaired two-tailed t-test; Supplementary Figure S4).

As Lonafarnib has previously been shown to increase the activation of lysosomes (Hernandez et al., 2019), we immunolabelled for lysosomal associated membrane protein 1 (LAMP1) in 3xTg AD mice. LAMP1+ neurons in dSub were more prominent in Lonafarnib infused (*n* = 4), compared to vehicle infused mice (*n* = 8), and overlapped with fibrillar OC + amyloid plaques (Supplementary Figure S5).

### Context-dependent spatial memory testing

The basic training and testing protocol of the context-dependent spatial memory task (Supplementary Figure S6) is described in (Julian et al., 2015). In brief, starting 5 days before the experiment, animals were taught to dig in a brain cup for a food reward (Weetos choco, Nestlé S.A.) in their home cage by providing them once daily with the reward gradually buried deeper under ginger-scented bedding (1 g of ginger for every 100 g of bedding) while being gradually food deprived to maintain 90–95% of their free-feed weight.

Disoriented mice were trained to dig for buried food rewards in two different chambers, one with square boundaries (4 × 29.25 cm) and one with circle boundaries (157 cm circumference). All chambers were built out of rectangular Legos (2 × 1 cm; Lego A/S, Billund, Denmark), and were 15 cm tall. Rewards were buried under ginger-scented bedding in cups embedded in the chamber floors. Each chamber was surrounded by the same distal cues for orientation. There were four possible reward locations in each chamber, and the rewarded location differed between the square- and circle-chamber relative to the common reference frame provided by the distal cues. Pilot experiments revealed that mice could successfully discriminate the square and circle reward locations above chance after 8 trials. Therefore, the training phase consisted of four training trials per chamber per day for 2 days, with successive trials alternated across chambers (8 trials total in the square-chamber and 8 trials total in the circle-chamber). If a given mouse achieved 66.6% correct performance during training, contextual memory was then tested in 4 testing sessions across 4 days, with 8 trials per session.

During each testing session, the first two trials consisted of spatial memory being tested in the square- and circle-chamber with rewards. In trials 3-6, spatial memory was tested in four chambers with morphed boundary geometry, which continuously ranged from most-square-like to most-circle-like: a pentagon (5 × 31.4 cm), a hexagon (6 × 26.16 cm), an octagon (8 × 19.6 cm), and a decagon (10 × 15.7 cm). During the final two trials of each testing session (trials 7-8), the animals were again tested in the square- and circle-chambers with rewards. The order of the square-, circle-, and morphed-chambers across trials in each session was randomized but was the same for each animal on a given day’s session. If a given mouse achieved 66.6% correct performance during testing, contextual memory was tested in an ambiguous half-square half-circle context (the “Squircle”) (Julian and Doeller, 2021).

During reward trials, mice were removed from the apparatus and the trial ended after they had found the reward. During unrewarded trials, they were removed, and the trial ended after their first dig, or after 5 min (whichever came later). Chambers were cleaned with ethanol after each trial to remove odor trails. Dig locations and time spent in these locations were calculated using ANY-maze video tracking system (Stoelting Europe) via an overhead, centrally located camera (DMK 22AUC03 USB 2.0 monochrome industrial camera, The imaging Source Europe, Germany).

### Proteomic analysis of amyloid-β and tau concentrations in cerebrospinal fluid

The MILLIPLEX® MAP human Aβ and tau magnetic bead panel 4-plex ELISA kit (Millipore, Burlington, MA, United States) and the Bio-Plex 200 System instrument (Biorad, Hercules, CA, United States) were used to assess simultaneously the concentrations of Aβ_40_, Aβ_42_, total tau (t-tau), and phosphorylated tau at Thr181 (p-tau) in CSF samples. The samples were undiluted and analyzed in duplicates. The lower limit of quantification (LLOQ) for each protein is shown in Supplementary Figure S7.

### Tissue processing and immunohistochemistry

Mice were administered a lethal dose of sodium pentobarbital (100 mg/ml; Apotekforeningen, Oslo, Norway) and transcardially perfused with Ringer’s solution followed by paraformaldehyde (PFA, 4%; Sigma-Aldrich) in 125 mM phosphate buffer (PB). Brains were extracted and fixed for a minimum of 24 h in PFA at 4°C and transferred to a 2% DMSO solution prepared in PB for 24 h at 4°C. Brains were sectioned coronally at 40 µm on a freezing-sliding microtome (Microm HM430, ThermoFisher Scientific, Waltham, MA, United States). An incision was made in the non-implanted hemisphere for visualization of the control hemisphere. Immunohistochemical processing was conducted on tissue, see (Bjorkli, 2019; Bjorkli and Lagartos-Donate, 2022) for detailed protocols, and Supplementary Table S1 for key resources.

Previous research has indicated that a differential microtubule-associated protein 2 (MAP2) immunolabeling pattern can distinguish dense-core from diffuse amyloid plaques using DAB (Sigma-Aldrich, St. Louis, MO, United States) as a chromogen (D'Andrea and Nagele, 2002). See (Bjorkli, 2022) for detailed protocol.

One series of each brain was dehydrated in ethanol, cleared in xylene (Merck Chemicals, Darmstadt, Germany) and rehydrated before staining with Cresyl violet (Nissl; 1 g/L) for 3 min to verify probe placement. The sections were then alternatively dipped in ethanol–acetic acid (5 ml acetic acid in 1 L 70% ethanol) and rinsed with cold water until the desired differentiation was obtained, then dehydrated, cleared in xylene and coverslipped with entellan containing xylene (Merck Chemicals).

Sections were scanned using a Mirax-midi slide scanner (objective 20X, NA 0.8; Carl Zeiss Microscopy, Oberkochen, Germany), using either reflected fluorescence (for sections stained with a fluorophore) or transmitted white light (for sections immunolabelled with Nissl DAB, or Gallyas-silver) as the light source.

### Quantification of intraneuronal amyloid-β and tau, and amyloid plaques.

Series of sections were chosen randomly and coded to ensure blinding to the investigators. The number of cells containing intraneuronal Aβ, tau, and amyloid plaques, in dSub and LEC of 3xTg AD mice infused with a vehicle or drugs was estimated with Ilastik using the Density Cell Counting workflow (Berg et al., 2019). dSub and LEC was delineated using cytoarchitectonic features in sections stained with Nissl, based on The Paxinos & Franklin Mouse Brain Atlas (Paxinos and Franklin, 2004). The same surface area and rostrocaudal levels of each brain region was selected, and at least 4 brain sections were used for each infused hemisphere. Damaged regions of brain sections were excluded from analyses to avoid false-positive antibody expression.

### Statistics

Effect size (Cohen’s D) was calculated based on initial experiments between animals infused with Fasudil and animals infused with a vehicle, and the resulting effects on intraneuronal Aβ in dSub. Based on each group consisting of *n* = 2 animals, an effect size of 0.75 was calculated (0.8 is considered a large effect size) (Cohen, 1992). Most of the dataset was normally distributed (Shapiro-Wilk test) and therefore two-tailed, unpaired t-tests were used to compare mean differences. For the minor parts of the dataset that was not normally distributed, nonparametric statistical tests were used (Mann-Whitney U). Statistical comparisons of behavioral data across vehicle and drug infused mice were conducted based on trial-wise pooling of data across mice separately for each group. Behavioral performance in the morphed environments of the context-dependent spatial memory task (Supplementary Figure S6) was calculated as follows: dig in square-consistent location = 1, dig in circle-consistent location = 0, dig in any other location = 0.5. Context-consistency of reward locations was determined relative to the common reference frame defined by the distal cues shared across all contexts. We then assessed whether performance in the morphed environments was associated with more context-appropriate choices for animals treated with a vehicle compared to drugs. All statistical tests and graphs were made in Prism 9 (GraphPad Software Inc., CA, United States).

## Results

### Fasudil treatment attenuated amyloid-β and tau pathology in early and late phases of the disease

**FIGURE 1:**
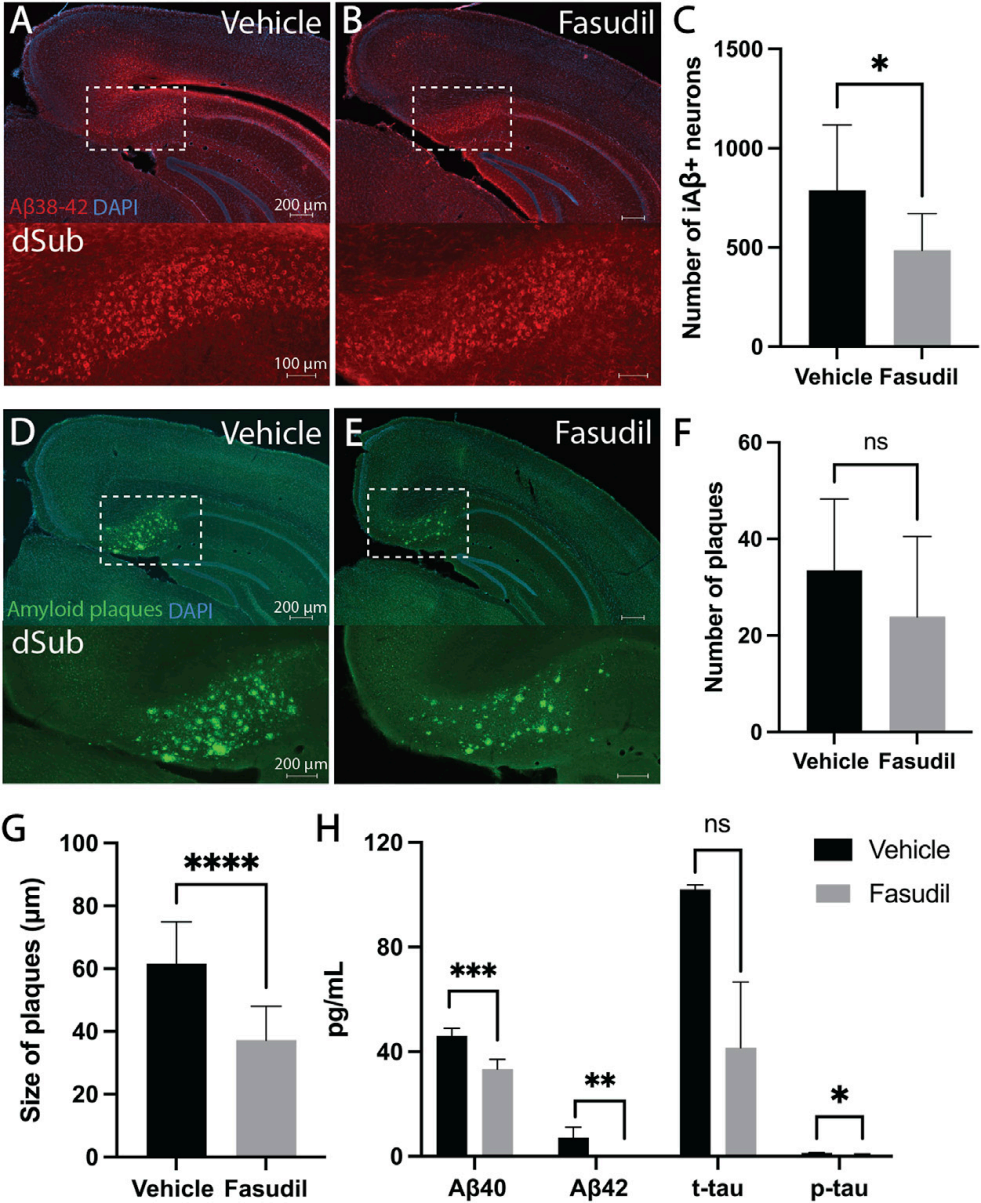
Inhibiting the Wnt-PCP pathway attenuated amyloid-β and tau pathology in early and late phase AD. **(A)** Intraneuronal Aβ_38-42_ (McSA1; red) in dSub in 3xTg AD mice (*n* = 4) receiving infusions of a vehicle (i.e., saline). DAPI counterstain (blue). **(B)** Aβ_38-42_ (McSA1; red) immunoreactivity in dSub in 3xTg AD mice (*n* = 6) receiving infusions of Fasudil. DAPI counterstain (blue). **(C)** Mean number of Aβ+ neurons in dSub of 3xTg AD mice after infusions of a vehicle and Fasudil. Intraneuronal Aβ in dSub was quantified from at least 7 brain sections for each animal using Ilastik. Error bars denote ±1 SD, unpaired two-tailed *t*-test, *p < 0.05. **(D)** Amyloid plaques (amyloid fibrils OC; green) in dSub of 3xTg AD mice (*n* = 2) receiving infusions of a vehicle. DAPI counterstain (blue). **(E)** Amyloid plaques (amyloid fibrils OC; green) in dSub of 3xTg AD mice (*n* = 4) receiving infusions of Fasudil. DAPI counterstain (blue). **(F)** Mean number of amyloid plaques in dSub of 3xTg AD mice after infusions of a vehicle and Fasudil. Amyloid plaques in dSub were quantified from at least 4 brain sections for each animal using Ilastik. Error bars denote ±1 SD, unpaired two-tailed *t*-test, n. s. non-significant. **(G)** Mean size of amyloid plaques in dSub of 3xTg AD mice after infusions of a vehicle and Fasudil. Amyloid plaques in dSub were quantified from at least 4 brain sections for each animal using Ilastik. Error bars denote ±1 SD, unpaired two-tailed *t*-test, ****: *p* < 0.0001. **(H)** Mean concentrations of duplicates of Aβ_40_, Aβ_42_, p-tau and t-tau CSF levels (pg/ml) as measured by multiplex ELISA on the last day of infusion (day 14) of a vehicle (*n* = 6) or Fasudil (*n* = 10) in young and older 3xTg AD mice. Error bars denote ±1 SD, unpaired two-tailed *t*-test, ***: *p* < 0.001, **: *p* < 0.01, *: *p* < 0.05. Abbreviations: Aβ, amyloid-β; dSub, dorsal subiculum; iAβ, intraneuronal Aβ; t-tau, total tau; p-tau, phosphorylated tau.

First, we wanted to assess whether Fasudil (administered for a duration of 14 days) would affect intraneuronal Aβ which is present already at 1-month-of-age in 3xTg AD mice (Supplementary Figure S8A; Supplementary Table S3). Intraventricular administration of Fasudil in 6 months-old 3xTg AD mice reduced the number of Aβ+ neurons in dSub as compared to vehicle infused mice (t_18_ = 2.63, *p* = 0.0169, unpaired two-tailed t-test; Figures 1A–C). We then went on to assess the effect of Fasudil on amyloid plaques in 3xTg AD mice, which accumulate at 13-months-of-age (Supplementary Figures S8B,C; Supplementary Table S3). In older 3xTg AD mice (14 months-old), Fasudil infusions moderately reduced the number of dense-core amyloid plaques (Supplementary Figure S9) in dSub as compared to vehicle infused mice (n.s.; Figures 1D–F). The size of amyloid plaques, on the other hand, was effectively reduced in Fasudil, compared to vehicle, treated mice (t_26_ = 4.69, *p* < 0.0001, unpaired two-tailed t-test; Figures 1D,E,G). We went on to conduct proteomic analyses to assess CSF Aβ and tau levels following Fasudil treatment (Figure 1H). On average across younger and older mice, Fasudil effectively reduced CSF Aβ_40_ (t_6_ = 5.96, *p* < 0.001, unpaired two-tailed t-test) and Aβ_42_ levels (t_5_ = 5.59, *p* < 0.01, unpaired two-tailed t-test). Fasudil treatment also effectively reduced CSF p-tau levels (t_5_ = 2.86, *p* < 0.05, unpaired two-tailed t-test), and moderately reduced CSF t-tau levels (n.s.).

### Lonafarnib treatment attenuated amyloid-β and tau pathology in early and late phases of the disease

**FIGURE 2:**
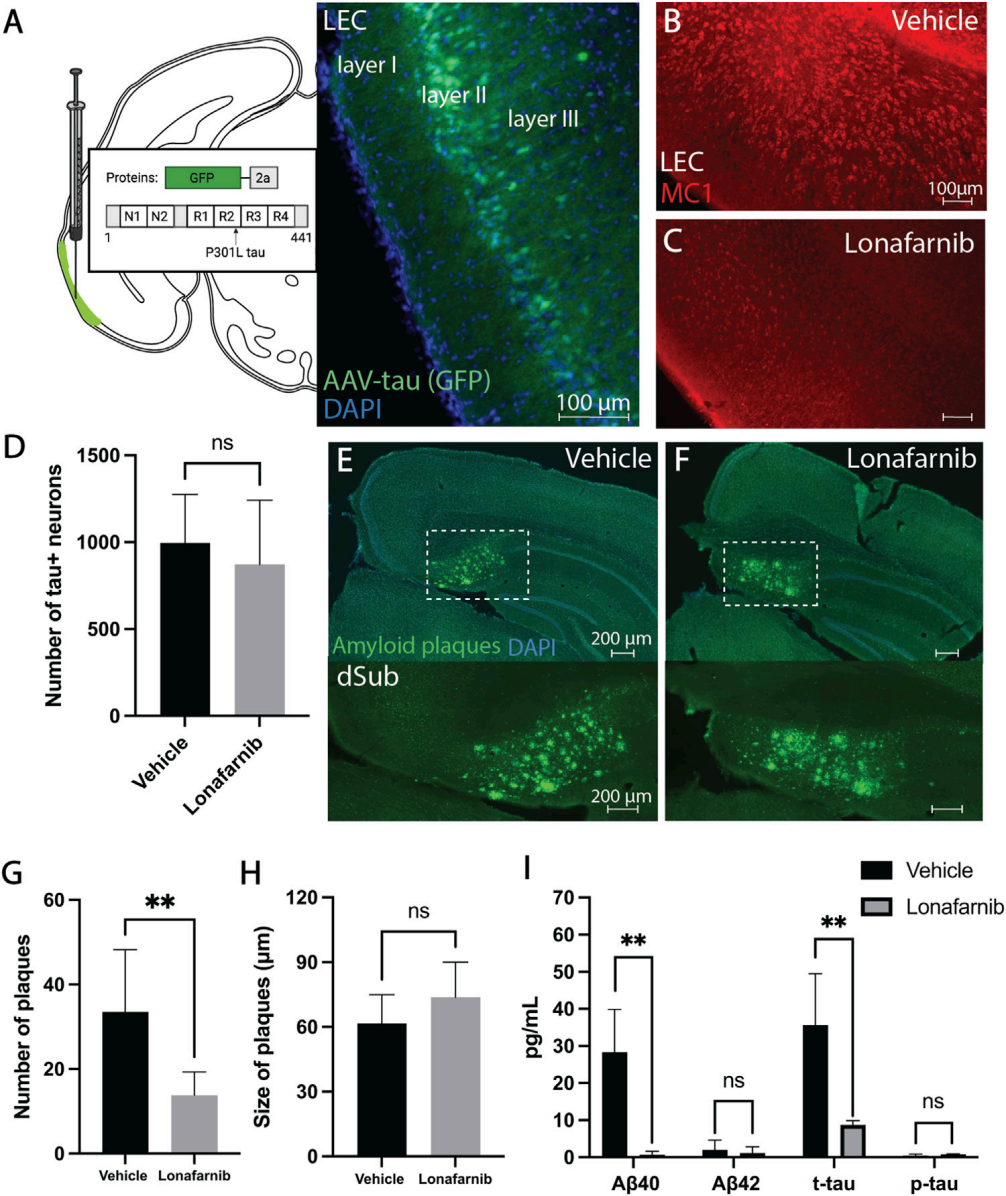
Induction of autophagy attenuated amyloid-β and tau pathology in early and late phase AD. **(A)** Schematic for the injection of AAV8 GFP-2a-P301Ltau (AAV-tau) into LEC layer II (GFP; green) of 3xTg AD mice (*n* = 4), and the proteins encoded in the viral construct. DAPI counterstain (blue). **(B)** Conformation-specific tau (MC1; red) in LEC of 3xTg AD mice (*n* = 2) receiving infusions of a vehicle (i.e., saline). **(C)** Conformation-specific tau (MC1; red) in LEC of 3xTg AD mice (*n* = 2) receiving infusions of Lonafarnib. **(D)** Mean number of tau + neurons in LEC of 3xTg AD mice after infusions of a vehicle and Lonafarnib. Intraneuronal tau in LEC layer II was quantified from at least 4 brain sections for each animal using Ilastik. Error bars denote ±1 SD, unpaired two-tailed *t*-test, n.s.: non-significant. **(E)** Amyloid plaques (amyloid fibrils OC; green) in dSub of 3xTg AD mice (*n* = 2) receiving infusions of a vehicle. DAPI counterstain (blue). **(F)** Amyloid plaques (amyloid fibrils OC; green) in dSub of 3xTg AD mice (*n* = 2) receiving infusions of Lonafarnib. DAPI counterstain (blue). **(G)** Mean number of amyloid plaques in dSub of 3xTg AD mice after infusions of a vehicle and Lonafarnib. Amyloid plaques in dSub were quantified from at least 4 brain sections for each animal using Ilastik. Error bars denote ±1 SD, unpaired two-tailed *t*-test, **: *p* < 0.01. **(H)** Mean size of amyloid plaques in dSub of 3xTg AD mice after infusions of a vehicle and Lonafarnib. Amyloid plaques in dSub were quantified from at least 4 brain sections for each animal using Ilastik. Error bars denote ±1 SD, unpaired two-tailed t-test, n.s.: non-significant. **(I)** Mean concentrations of duplicates of Aβ_40_, Aβ_42_, p-tau and t-tau CSF levels (pg/ml) as measured by multiplex ELISA on the last day of infusion (day 10) of a vehicle (*n* = 8) or Lonafarnib (*n* = 4) in young (injected with AAV-tau) and older 3xTg AD mice. Error bars denote ±1 SD, unpaired two-tailed t-test, **: *p* < 0.01. Abbreviations: GFP, green fluorescent protein; LEC, lateral entorhinal cortex; AAV, adeno-associated virus; dSub, dorsal subiculum; t-tau, total tau; p-tau, phosphorylated tau.

Similarly, we also wanted to assess whether Lonafarnib (administered for a duration of 10 days) would affect AD-related neuropathology present in early phases of AD. Since previous research suggests that Lonafarnib can reduce tau levels, we assessed not only early intraneuronal Aβ but also early tau abnormalities following infusions. Lonafarnib did not affect the number of Aβ+ neurons in dSub in young 3xTg AD mice (Supplementary Figure S10). Since the earliest accumulation of non-fibrillar tau (recognized by the MC1 antibody) is not present in 3xTg AD mice until around 9-months-of-age in CA1 (Supplementary Figure S11; Supplementary Table S3 **)**, we overexpressed human tau in LEC layer II (an area that is early involved in NFT deposition in AD patients), of 6 months-old 3xTg AD mice (Figure 2A). Lonafarnib infusions moderately reduced the number of tau+ neurons in LEC following injection of AAV-tau (n.s.; Figures 2B–D).

In older 3xTg AD mice (14 months-old), Lonafarnib infusions effectively reduced the number of dense-core amyloid plaques that overlapped with MC1 immunolabelling (Supplementary Figures S9, S12) in dSub as compared to vehicle infused mice (t_14_ = 3.86, *p* = 0.0017, unpaired two-tailed *t*-test; Figures 2E–G). The size of amyloid plaques, on the other hand, was moderately increased in Lonafarnib, compared to vehicle, treated mice (n.s.; Figures 2E,F,H). We then conducted proteomic analyses to assess CSF Aβ and tau levels following Lonafarnib treatment (Figure 2I). On average, across younger (injected with AAV-tau) and older mice, Lonafarnib effectively reduced CSF Aβ_40_ (t_6_ = 4.53, *p* < 0.01, unpaired two-tailed *t*-test) and t-tau levels (t_6_ = 4.05, *p* < 0.01, unpaired two-tailed t-test). Lonafarnib treatment moderately reduced CSF Aβ_42_ (n.s.) and p-tau levels (n.s.).

### Combinatorial drug treatment effectively attenuated Alzheimer’s disease-related pathology at the molecular and functional level

**FIGURE 3:**
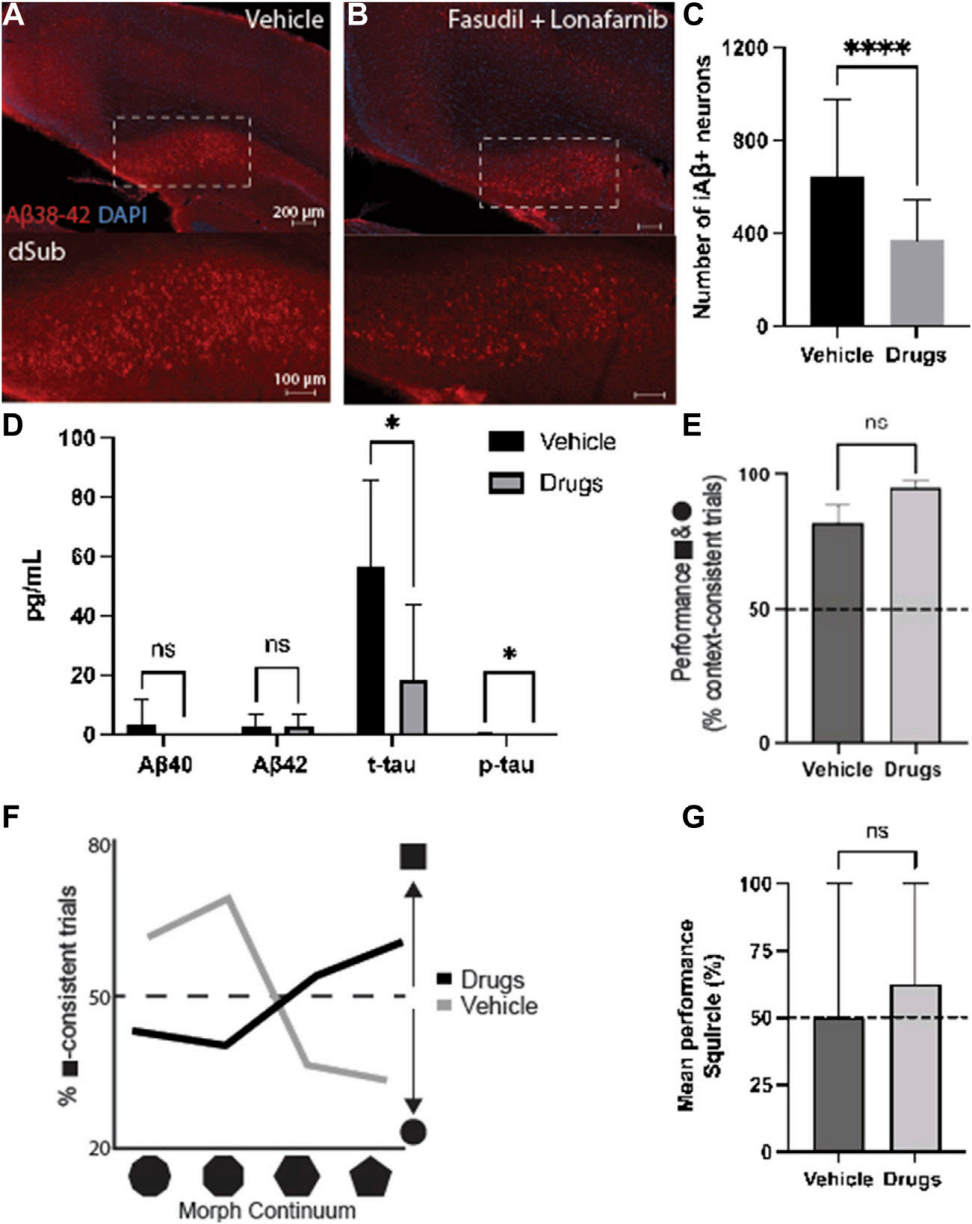
Combinatorial targeting of Wnt and mTOR pathways effectively attenuates neuropathology and context-dependent spatial memory deficits. **(A)** Intraneuronal Aβ_38-42_ (McSA1; red) in dSub in 3xTg AD mice (*n* = 4) receiving infusions of a vehicle (i.e., saline). DAPI counterstain (blue). **(B)** Aβ_38-42_ (McSA1; red) immunoreactivity in dSub in 3xTg AD mice (*n* = 4) receiving infusions of Fasudil and Lonafarnib. DAPI counterstain (blue). **(C)** Mean number of Aβ+ neurons in dSub of 3xTg AD mice after infusions of a vehicle, or Fasudil and Lonafarnib. Intraneuronal Aβ in dSub was quantified from at least 7 brain sections for each animal using Ilastik. Error bars denote ±1 SD, unpaired two-tailed *t*-test, ****p < 0.0001. **(D)** Mean concentrations of duplicates of Aβ_40_, Aβ_42_, p-tau and t-tau CSF levels (pg/ml) as measured by multiplex ELISA on the last day of infusion (day 7) of a vehicle (*n* = 6) or Fasudil and Lonafarnib (*n* = 4) in young 3xTg AD mice. Error bars denote ±1 SD, unpaired two-tailed *t*-test, *p < 0.05. **(E)** Mean behavioral performance (% of trials with context-appropriate choices) in 3xTg AD mice infused with a vehicle (*n* = 3) or Fasudil and Lonafarnib (*n* = 4) in the square- and circle-chambers. Error bars denote ±1 SD, unpaired two-tailed *t*-test, n.s.: non-significant. **(F)** Percentage of trials with square context-consistent digs in 3xTg AD mice infused with a vehicle (*n* = 3) or Fasudil and Lonafarnib (*n* = 4) in each of the four morphed environments, ranging from most circle-like to most square-like in boundary geometry shape. **(G)** Mean behavioral performance (% of trials with context-appropriate choices) in 3xTg AD mice infused with a vehicle (*n* = 3) or Fasudil and Lonafarnib (*n* = 4) in the Squircle context. Error bars denote ±1 SD, unpaired two-tailed t-test, n.s.: non-significant. Abbreviations: Aβ, amyloid-β; dSub, dorsal subiculum; iAβ, intraneuronal Aβ; t-tau, total tau; p-tau, phosphorylated tau.

Since both drugs appeared to reduce AD-related neuropathology, we wanted to assess their combinatorial effects when administered for 7 days during earlier phases of the disease (4-months-of-age). Since these mice were young, the treatment effects on intraneuronal Aβ in dSub were assessed, without the possibility of assessing amyloid plaques or tau pathology (Supplementary Table S3). Combinatorial treatment of drugs effectively reduced the number of Aβ+ neurons in dSub of mice (t_80_ = 4.66, *p* < 0.0001, unpaired two-tailed *t*-test; Figures 3A–C).

Proteomic analyses of CSF Aβ and tau levels (Figure 3D) revealed that on average, combinatorial treatment effectively reduced CSF t-tau (t_10_ = 2.39, *p* < 0.05, unpaired two-tailed *t*-test) and p-tau levels (t_10_ = 2.24, *p* < 0.05, unpaired two-tailed *t*-test). Combinatorial treatment moderately reduced CSF Aβ_40-42_ levels (n.s.).

We then examined context-dependent spatial memory (Supplementary Figure S6) in 3xTg AD mice at 4-months-of-age, the same age as when cognitive deficits usually begin in this model (Billings et al., 2005), after vehicle and drug infusions. Mice were trained to search for buried food rewards in two different contexts, one square and one circle (more details can be found in (Julian et al., 2015)). Animals infused with drugs initially searched in context-appropriate reward locations slightly more often than those infused with a vehicle. (n.s.; Figure 3E). We further examined contextual memory recall in environments with progressive morphs of boundary geometry, increasing the number of boundary walls from most square-like to most circle-like. Compared to animals infused with a vehicle, drug infused mice searched more often in context-consistent reward locations (χ^2^ = 69.17, *p* < 0.0001; Pearson’s Chi-squared test; Figure 3F). Furthermore, when contextual memory was tested in an ambiguous half-square half-circle context (the “Squircle”), mice infused with drugs were slightly more likely to dig at a location previously associated with either the square or circle, compared to locations not associated with any context, and compared to control animals infused with a vehicle (n.s.; Figure 3G). Thus, combinatorial drug treatment led to an improvement of cognitive deficits usually associated with canonical AD.

## Discussion

In this study we repurposed two FDA-approved drugs, Fasudil and Lonafarnib, both targeting independent biochemical cascades that halted the development of AD pathology in 3xTg AD mice. Treatment with Fasudil reduced early intraneuronal Aβ, the number and size of amyloid plaques in dSub, and CSF Aβ_40-42_ and p-tau levels. Lonafarnib infusions, on the other hand, did not affect intraneuronal Aβ but rather reduced early non-fibrillar forms of tau after overexpression in LEC layer II. Treatment with Lonafarnib also reduced the number of amyloid plaques, but unexpectedly increased their size in dSub, and only effectively decreased CSF Aβ_40_ and t-tau levels. Both drugs affected dense-core, rather than diffuse, amyloid plaques, and the former is associated with microglial activation, neurodegeneration, and cognitive decline in AD patients (Knowles et al., 1999; DeTure and Dickson, 2019). We found that novel combinatorial administration of these drugs effectively reduced early intraneuronal Aβ in younger mice, led to reduced CSF Aβ_40_ and p- and t-tau levels, and improved context-dependent spatial memory. This type of pattern completion is thought to depend on the normal function of the hippocampus (Pilly et al., 2018; Hainmueller and Bartos, 2020), a region that is compromised during early phases of AD.

Our results following Fasudil infusions are in line with previous reports of its efficacy in reducing Aβ levels (Tang and Liou, 2007; Herskowitz et al., 2013; Elliott et al., 2018; Sellers et al., 2018). Similarly, our findings after Lonafarnib treatment coincide with previous results of its efficacy to reduce AD-related neuropathology (Hernandez et al., 2019). Our findings of larger amyloid plaques following Lonafarnib treatment may be explained by reports of diminished levels of regulatory proteins in the autophagic system in older ages (Nixon and Yang, 2011; Menzies et al., 2017), which may lead to increased sequestration of nearby Aβ peptides by amyloid plaques. Conversely, this finding could be due to an increase in intracellular digestion, resulting in a larger expansion of amyloid plaques (D'Andrea et al., 2001).

The efficacy of the novel combinatorial treatment of both drugs presented here aligns with several previous findings. Aβ has not only been shown to activate Wnt-PCP signaling through Dkk1 (Bafico et al., 2001; Sellers et al., 2018), but also to increase mTOR activity in 3xTg AD mice brain regions with high levels of intraneuronal Aβ (Caccamo et al., 2010; Caccamo et al., 2011). Activation of Wnt-PCP also leads to induction of GSK-3β which contributes to the formation of tau pathology (Moon et al., 2004), and which has been found to suppress autophagy via mTOR complex 1 (mTORC1) and lysosomes (Azoulay-Alfaguter et al., 2015). Thus, regulating the tightly-linked Wnt and mTOR pathways (Yu et al., 2007) at early stages of AD has beneficial therapeutic effects in AD mouse models.

### Limitations of the study

There are potential limitations of this study that should be considered while interpreting our results. We found that CSF t-tau levels generally increased in the first few days of microdialysis sampling, with lower, but still elevated levels at the end of drug treatment. This is consistent with CSF t-tau concentrations increasing in the first few days following injury (i.e., probe implantation in our experiments), then reduction over time (Neselius et al., 2012). However, we have previously shown that long-term implantation of our microdialysis probes does not lead to observable neuroinflammation in 3xTg AD mice (Bjorkli et al., 2021). In addition, intracerebral microdialysis is an invasive technique, so it is currently only used in patients requiring neurocritical care, neurosurgery, or brain biopsy (Marklund et al., 2009). A leaky BBB has often been found to precede amyloid plaque formation in AD patients (Zenaro et al., 2017) and 3xTg AD mice (Ujiie et al., 2003), and could therefor affect drug penetration in our mice. Moreover, in our study and most preclinical translational AD research, there is an increased focus on familial rather than sporadic forms of AD. This limitation, however, should not detract from the value of the current study and other studies using transgenic models, as the continuums of sporadic and familial forms of AD are very similar, except for age-of-onset (Holmes and Lovestone, 2002).

### Conclusion and future directions

In this study, we repurposed, Fasudil and Lonafarnib, targeting synaptic formation and autophagic pathways respectively, to test their therapeutic potential for attenuating AD-related pathology. We show that combinatorial treatment with both drugs was effective at reducing intraneuronal Aβ and led to improved cognitive performance in mice. Importantly, based on our own characterization of 3xTg AD mice, we demonstrate potential timepoints for when it would be beneficial during the disease continuum to target Wnt and autophagic pathways to attenuate AD pathology. Targeting Wnt during early and late stages of the disease effectively reduced Aβ, whereas targeting mTOR did not affect Aβ levels at early stages of AD. Overexpression of tau in LEC layer II was effectively reduced by Lonafarnib at early stages, but treatment led to an increase in size of amyloid plaques at later stages of AD. Combinatorial treatment of both drugs when 3xTg AD mice start to display cognitive impairments not only reduced intraneuronal Aβ, but also attenuated context-dependent spatial memory deficits. Therefore, future studies should aim for longitudinal combinatorial treatment starting before observable cognitive deficits, while examining the effect on AD pathophysiology at later stages of the disease. Taken together, our findings lend support to the application of Wnt and mTOR regulation to attenuate AD pathology at various therapeutic timepoints in preclinical models.

## Data Availability

The original contributions presented in the study are included in the article/Supplementary Material, further inquiries can be directed to the corresponding author.

## Footnotes

1 Wnt is a portmanteau created from the names of two genes, the Drosohila wingless gene and the murine homologue integration site 1 gene.

2 Farnesylation is a postttranslational modification of proteins.
