# Supplementary material for: Combined targeting of pathways regulating synaptic formation and autophagy attenuates Alzheimer’s disease pathology in mice
Source: Front Pharmacol. 2022 Aug 16;13:913971. doi: 10.3389/fphar.2022.913971 (PMC9426773; doi:10.3389/fphar.2022.913971)
Supplement: Supplementary file 1 [file Image12.pdf]

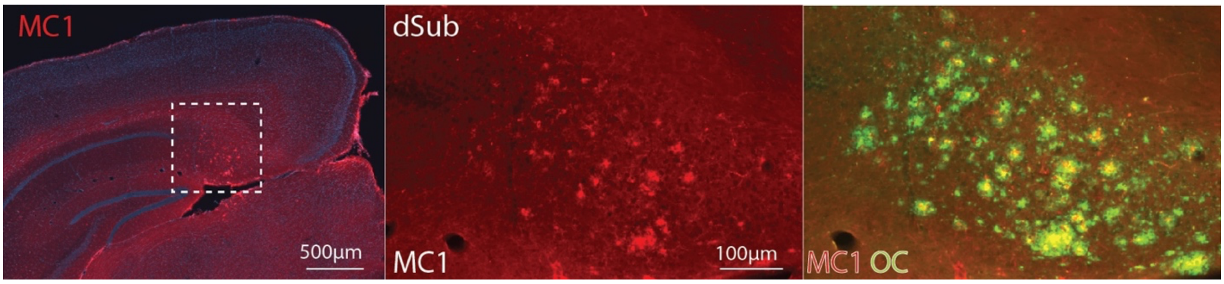

Supplementary Figure 12. MC1+ amyloid plaques in 3xTg AD mice. Conformation-specific tau (MC1; red) and fibrillar Aβ (OC; green) immunoreactivity in dSub at 13-months-of-age. Abbreviations; dSub: dorsal subiculum.
