# Supplementary material for: Combined targeting of pathways regulating synaptic formation and autophagy attenuates Alzheimer’s disease pathology in mice
Source: Front Pharmacol. 2022 Aug 16;13:913971. doi: 10.3389/fphar.2022.913971 (PMC9426773; doi:10.3389/fphar.2022.913971)
Supplement: Supplementary file 2 [file Image5.pdf]

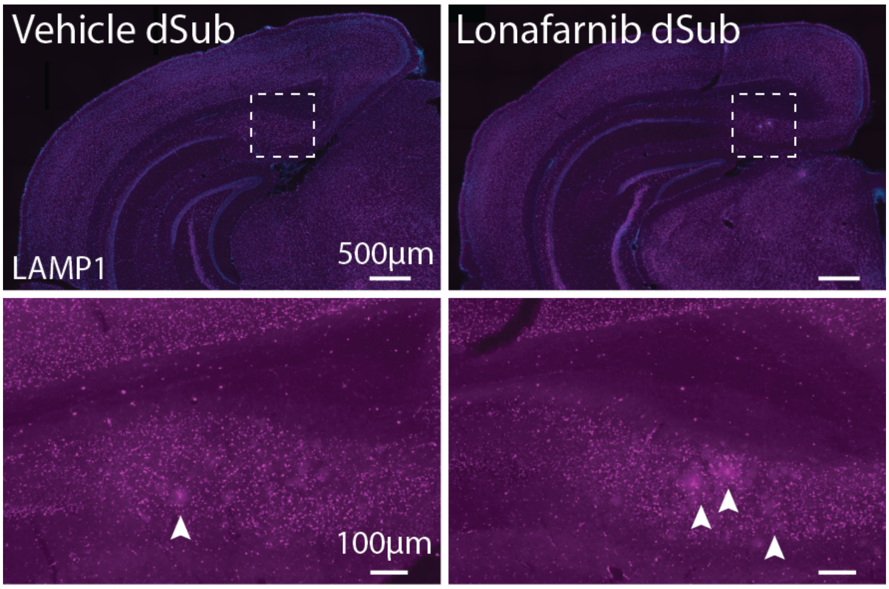

Supplementary Figure 5. LAMP1 (lysosomal marker; purple) immunoreactivity (arrowheads) in dSub in 14-month-old 3xTg AD mice receiving infusions of a vehicle (n = 2) or Lonafarnib (n = 4). Abbreviations; dSub: dorsal subiculum; LAMP1: lysosomal associated membrane protein 1.
