# Supplementary material for: Combined targeting of pathways regulating synaptic formation and autophagy attenuates Alzheimer’s disease pathology in mice
Source: Front Pharmacol. 2022 Aug 16;13:913971. doi: 10.3389/fphar.2022.913971 (PMC9426773; doi:10.3389/fphar.2022.913971)
Supplement: Supplementary file 3 [file Table1.pdf]

Supplementary Table 1: Key resources

Reagent type (species) or resource	Designation	Information	Identifiers/reference
**Strain, strain background (Mus musculus)**	3xTg AD	B6;129-Psen1^{tm1Mpm}Tg(APPSwe,tau P301L)1Lfa/Mmjax	MMRRC Strain #034830-JAX; RRID: MMRRC_034830-MU; PMID: 12895417
**Strain, strain background (Mus musculus)**	B6129	B6129SF2/J	Strain #:101045; RRID: IMSR_JAX:101045
**Genetic reagent (virus)**	AAV-CBA-GFP-2A-P301L-Tau (serotype 8)	Viral Vector Core at Kavli Institute for Systems Neuroscience; contact Dr. Raveendran, rajeevkumar.r.nair@ntnu.no	Gifted by Bradley Hyman's lab, Harvard Medical School; PMID: 31249873
**Antibody**	Mouse anti-Aβ (McSA1)*	Targets the N-terminal amino acids 1-12 of human Aβ	MediMabs Cat# MM-0015-1P, RRID:AB_1807985
**Antibody**	Anti-$A\beta_{42}$ (rabbit polyclonal)*	$A\beta_{42}$ (pre-oligomers)	Tecan (IBL) Cat# JP28051, RRID:AB_2341462
**Antibody**	Anti-oligomer A11 (rabbit polyclonal)	Soluble $A\beta_{40}$/oligomeric $A\beta_{42}$ (prefibrils)	Thermo Fisher Scientific Cat# AHB0052, RRID:AB_2536236
**Antibody**	Anti-amyloid fibrils OC (rabbit polyclonal)*	Amyloid fibrils/fibrillar oligomers (protofibrils)	Millipore Cat# AB2286, RRID:AB_1977024
**Antibody**	Anti-Iba1 (mouse monoclonal)	Ionized calcium binding adaptor molecule 1 (Iba1)	Abcam Cat# ab15690, RRID:AB_2224403
**Antibody**	Anti-TREM2 (rabbit monoclonal)	TREM2 receptor	Thermo Fisher Scientific Cat# MA5-30971, RRID:AB_2786636
**Antibody**	Anti-MAP2 (rabbit monoclonal)	Microtubule-associated protein 2	Abcam Cat# ab183830, RRID:AB_2895301; PMID: 12083391
**Antibody**	Anti-LAMP1 (rabbit polyclonal)	Lysosomal associated membrane protein 1	Sigma-Aldrich Cat# L1418, RRID:AB_477157
**Antibody**	Anti-phospho-tau AT8 (mouse monoclonal)	Tau phosphorylated at serine 202 and threonine 205	Thermo Fisher Scientific Cat# MN1020, RRID:AB_223647
**Antibody**	Anti-tau HT7 (mouse monoclonal)	Recognized $tau_{159-163}$ and does not cross-react with murine tau	Thermo Fisher Scientific Cat# MN1000, RRID:AB_2314654; PMID: 1729400
**Antibody**	Anti-tau MC1 (mouse monoclonal)	Conformation specific, detects misfolded tau relevant to tauopathy	Gifted by Peter Davies, Department of Pathology, Albert Einstein College of Medicine
**Antibody**	Anti-NeuN (rabbit monoclonal)	Neuronal labelling	Abcam Cat# ab177487, RRID:AB_2532109
**Antibody**	Goat anti-mouse IgG (AF 657)	Secondary antibody	Thermo Fisher Scientific Cat# A-21235, RRID:AB_2535804
**Antibody**	Goat anti-mouse IgG (AF 546)	Secondary antibody	Thermo Fisher Scientific Cat# A-11030, RRID:AB_2534089

**Antibody**	Goat anti-mouse IgG (AF 488)	Secondary antibody	Thermo Fisher Scientific Cat# A28175, RRID:AB_2536161
**Antibody**	Goat anti-rabbit IgG (AF 488)	Secondary antibody	Molecular Probes Cat# A-11008, RRID:AB_143165
**Antibody**	Goat anti-rabbit IgG (AF 546)	Secondary antibody	Thermo Fisher Scientific Cat# A-11035, RRID:AB_2534093
**Chemical compound, drug**	Fasudil	Rho kinase inhibitor	Selleck Chemicals Cat# S1573; PMID: 29055813
**Chemical compound, drug**	Lonafarnib	Farnesyltransferase inhibitor with antitumor activity	Cayman Chemical Cat# CAY11746-1 mg; PMID: 30918111
**Chemical compound, drug**	DAPI (4',6-Diamidino-2-Phenylindole, Dihydrochloride)	Nuclear and chromosome counterstain	Thermo Fisher Scientific Cat# D1306, RRID:AB_2629482
**Chemical compound, drug**	Nissl (cresyl violet)	RNA labelling	Bjorkli 2019 doi: https://protocols.io/view/ihc-ad-neuropathology-protocol-btbmnik6.
**Chemical compound, drug**	Gallyas-silver staining	Modified silver impregnation of NFTs	Bjorkli & Lagartos-Donate 2022 doi: dx.doi.org/10.17504/protocols.io.b44cqysw.
**Chemical compound, drug**	DAB	Chromogen for detecting antibodies	Bjorkli 2022 doi: dx.doi.org/10.17504/protocols.io.b44fqytn.
**Software**	GraphPad Prism, version 9	Statistics and data visualization software	
**Software**	Zeiss ZEN lite	Microscope software	
**Software**	Ilastik	Cell counting software	PMID: 31570887
**Software**	ANY-maze – Stoelting Co.	Video tracking software	

* Labels amyloid plaques
