# Supplementary material for: Combined targeting of pathways regulating synaptic formation and autophagy attenuates Alzheimer’s disease pathology in mice
Source: Front Pharmacol. 2022 Aug 16;13:913971. doi: 10.3389/fphar.2022.913971 (PMC9426773; doi:10.3389/fphar.2022.913971)
Supplement: Supplementary file 4 [file Image9.pdf]

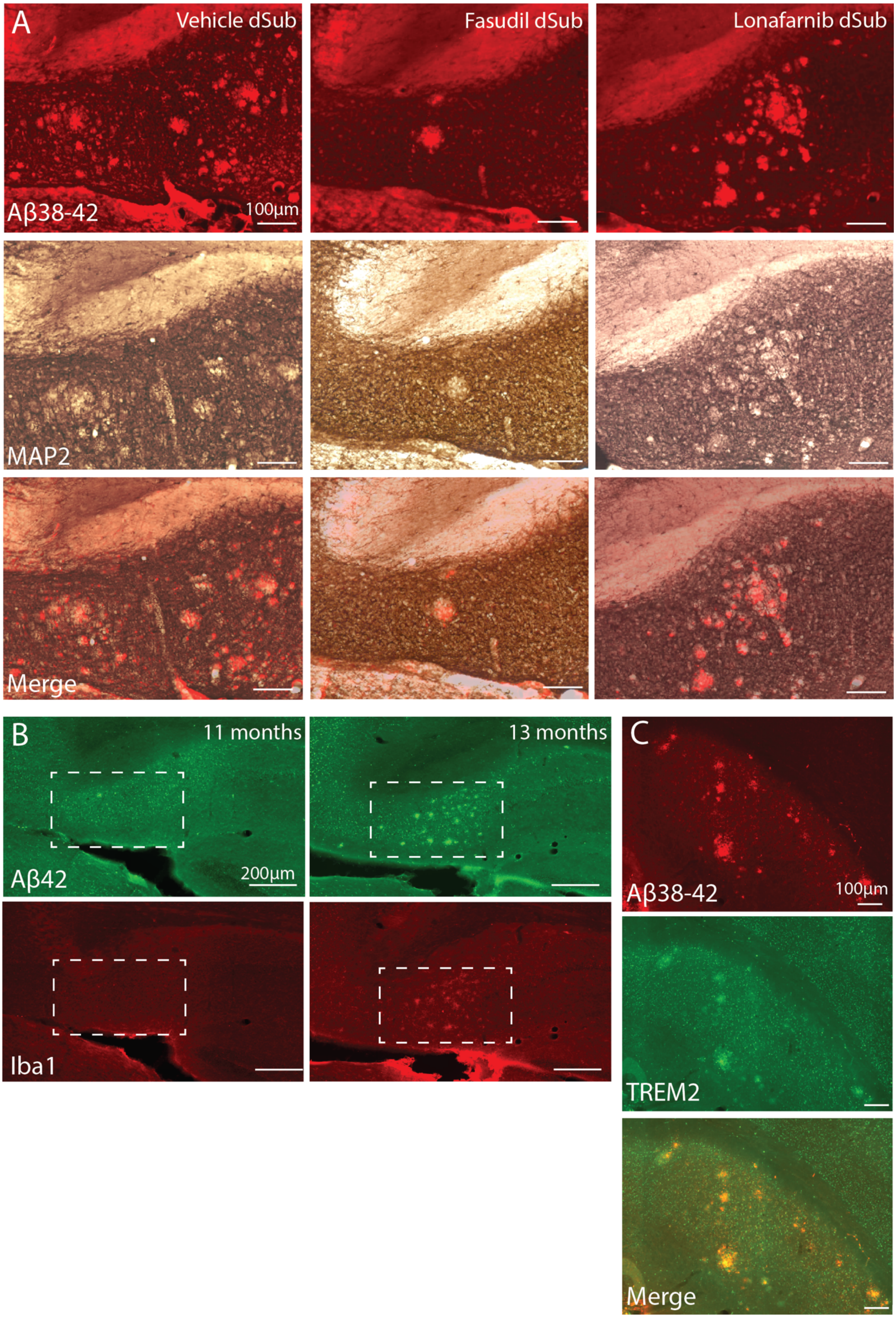

A
Vehicle dSub
Fasudil dSub
Lonafarnib dSub
Aβ38-42
100µm
MAP2
Merge
B
11 months
13 months
Aβ42
200µm
Iba1
C
Aβ38-42
100µm
TREM2
Merge

Supplementary Figure 9. Dense-core amyloid plaques after Fasudil and Lonafarnib infusions, and depiction of associated microglial activation surrounding dense-core amyloid plaques. (A) A$\beta_{38\text{-}42}$ (red; McSA1) immunolabelling for amyloid plaques (top panel) and DAB staining (brown) for microtubule-associated protein 2 middle panel; MAP2). McSA1 and the absence of MAP2 staining colocalized (bottom panel), confirming previous research indicating that MAP2 processes are absent within dense-core amyloid plaques. **(B)** Aβ_{42} (IBL Aβ_{42}; green) and Iba1 (microglial marker; red) immunoreactivity in dSub at 11- and 13-months-of-age in the 3xTg AD mouse model. **(C)** A$\beta_{38\text{-}42}$ (McSA1; red) and TREM2 (microglial receptor; green) immunoreactivity in dSub at 13-months-of-age. Abbreviations; Aβ: amyloid-β; dSub: dorsal subiculum; Iba1: Ionized calcium-binding adapter molecule 1; TREM2: triggering receptor expressed on myeloid cells 2.
