# Supplementary material for: Combined targeting of pathways regulating synaptic formation and autophagy attenuates Alzheimer’s disease pathology in mice
Source: Front Pharmacol. 2022 Aug 16;13:913971. doi: 10.3389/fphar.2022.913971 (PMC9426773; doi:10.3389/fphar.2022.913971)
Supplement: Supplementary file 5 [file Image10.pdf]

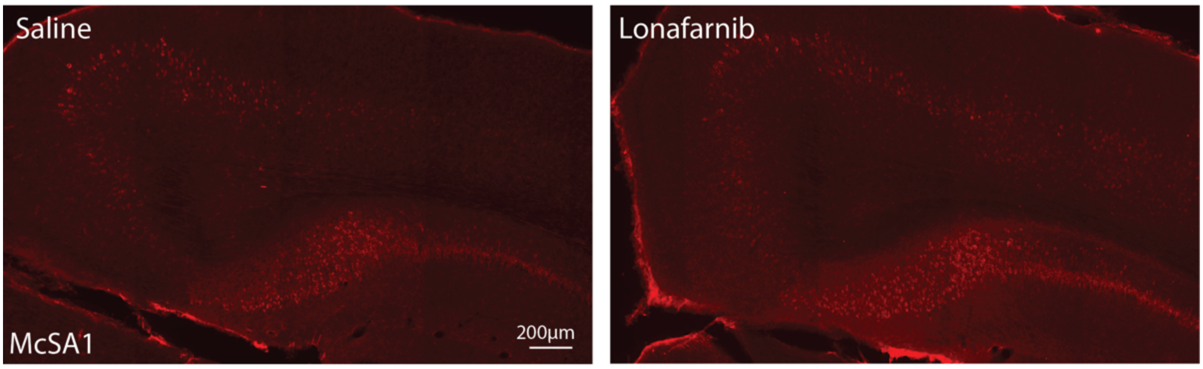

Supplementary Figure 10. Intraneuronal Aβ in dSub after Lonafarnib infusions. Lonafarnib did not affect the number of $A\beta_{38\text{-}42}$+ neurons (McSA1; red) in dSub in 6-month-old 3xTg AD mice (*n* = 2).
