# Supplementary material for: Combined targeting of pathways regulating synaptic formation and autophagy attenuates Alzheimer’s disease pathology in mice
Source: Front Pharmacol. 2022 Aug 16;13:913971. doi: 10.3389/fphar.2022.913971 (PMC9426773; doi:10.3389/fphar.2022.913971)
Supplement: Supplementary file 6 [file Table2.pdf]

Supplementary Table 2: Sample size for experimental conditions

Experimental condition	4 months-old	6 months-old	14 months-old
**Fasudil treatment (n)**		6	4
**Lonafarnib treatment (n)**		4*	2
**Combinatorial treatment (n)**	6^{+}		
**Saline (vehicle) treatment (n)**	5^{+}	6*	2

* 2 of these animals were injected with AAV-tau

$^{+}$ 3-4 of these animals underwent behavioral testing
