# Supplementary material for: Combined targeting of pathways regulating synaptic formation and autophagy attenuates Alzheimer’s disease pathology in mice
Source: Front Pharmacol. 2022 Aug 16;13:913971. doi: 10.3389/fphar.2022.913971 (PMC9426773; doi:10.3389/fphar.2022.913971)
Supplement: Supplementary file 7 [file Image6.pdf]

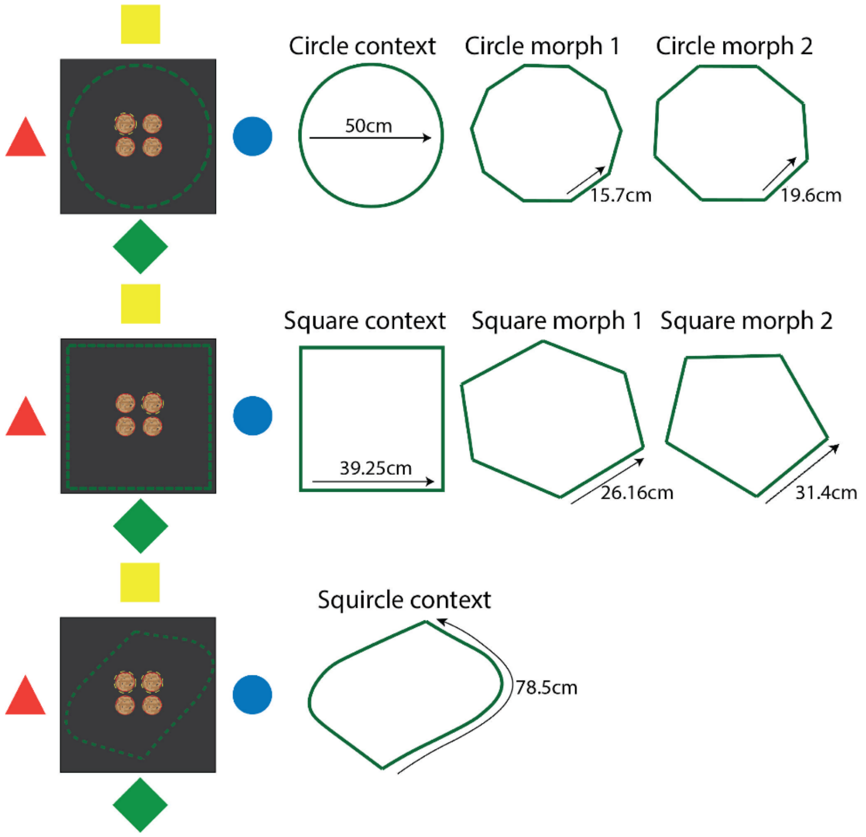

Supplementary Figure 6. Context-dependent spatial memory task design. Mice were initially taught to associate a specific reward location in a square- and a circle-chamber. The reward was buried in one of four cups with ginger-scented bedding. A trial was assessed as correct if the mice dug in the reward location associated with each chamber. If they passed the training phase (66.6 % correct digging), their contextual memory performance was tested in morph-chambers: a decagon (circle morph 1), an octagon (circle morph 2), a hexagon (square morph 1), and a pentagon (square morph 2). Mice were tested in morph-chambers for 4 days, with 8 sessions a day. If they were able to complete the morph testing, they were tested in a Squircle-chamber on the fifth day. Since the Squircle equally resembles the square- and a circle-chambers, a trial was considered correct if the mouse dug either of the reward locations attributable to the square- or circle-chamber. Figure adapted by permission from Nora Cecilie Ebbesen.
