# Supplementary material for: Combined targeting of pathways regulating synaptic formation and autophagy attenuates Alzheimer’s disease pathology in mice
Source: Front Pharmacol. 2022 Aug 16;13:913971. doi: 10.3389/fphar.2022.913971 (PMC9426773; doi:10.3389/fphar.2022.913971)
Supplement: Supplementary file 8 [file Image8.pdf]

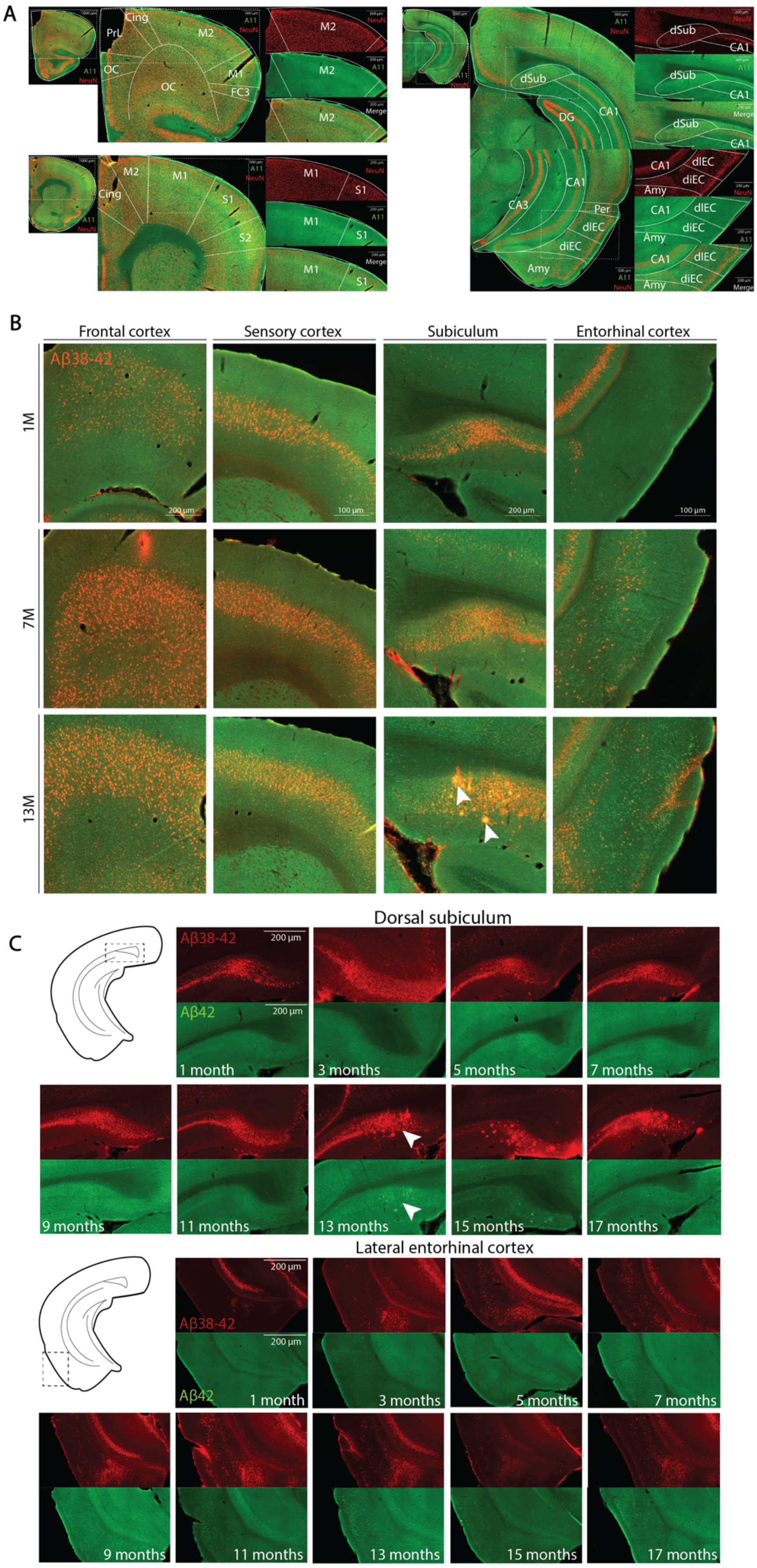
A
PrL
Cing
M2
OC
M1
FC3
A11
NeuN
Merge
S1
S2
dSub
CA1
DG
CA3
Per
dlEC
Amy
B
Frontal cortex
Sensory cortex
Subiculum
Entorhinal cortex
Aβ38-42
1M
7M
13M
200 µm
100 µm
C
Dorsal subiculum
Aβ42
1 month
3 months
5 months
7 months
9 months
11 months
13 months
15 months
17 months
Lateral entorhinal cortex

Supplementary Figure 8. Characterization of Aβ in the brain of our 3xTg AD mouse colony. (A) A11 (oligomeric Aβ specific; green) and NeuN (nuclei specific; red) immunoreactivity in the 3xTg AD mouse model at 1-month-of-age. At this age, there is little-to-none A11 immunoreactivity in frontal and sensory areas of the brain (left), as well as in the hippocampal and parahippocampal region (right). **(B)** $A\beta_{38-42}$ immunoreactivity in frontal cortex, sensory cortex, Sub, and EC in the 3xTg AD mouse model at 1, 7 and 13 months of age. $A\beta_{38-42}$ (McSA1; red) and $A\beta_{42}$ (IBL $A\beta_{42}$; green) immunoreactivity in the 3xTg AD mouse model. Amyloid plaques immunoreactive to $A\beta_{38-42}$ (McSA1 antibody) are first apparent at 13 months of age in subiculum. **(C)** $A\beta_{38-42}$ immunoreactivity in the 3xTg AD mouse model at various ages in the dSub and LEC. $A\beta_{38-42}$ (McSA1; red) and $A\beta_{42}$ (IBL $A\beta_{42}$; green) immunoreactivity in the 3xTg AD mouse model. According to the ABC scoring system, diffuse amyloid plaques is scored in the cerebral cortex, hippocampus, striatum, midbrain, brainstem, and cerebellum according to protocols established by Thal et al.[1] resulting in a Thal phase 0-5, which is translated into the NIA-AA score of A0-A3. Amyloid plaques immunoreactive to $A\beta_{38-42}$ (McSA1 antibody) are first apparent at 13 months of age in dSub, whereas plaques immunoreactive to $A\beta_{42}$ (IBL $A\beta_{42}$ antibody) are first apparent at 15 months of age in dSub. Abbreviations; S1: primary somatosensory cortex; S2: secondary somatosensory cortex; Olf: olfactory area; OC: orbital cortex; PrL: prelimbic cortex; Cing: cingulate cortex; M1: primary motor cortex; M2: secondary motor cortex; FC3: frontal cortex area 3; Ins: insular cortex; CA1-3: cornu ammonis field 1-3; Amy: amygdala; diEC: dorsal intermediate entorhinal cortex; dlEC: dorsolateral entorhinal cortex; dSub: dorsal subiculum; PER: perirhinal cortex. Aβ: amyloid-β; Sub: subiculum; LEC: lateral entorhinal cortex.

---

[1] Thal, D.R., Rub, U., Orantes, M., and Braak, H. (2002). Phases of A beta-deposition in the human brain and its relevance for the development of AD. *Neurology* 58**,** 1791-1800.
