# Supplementary material for: Combined targeting of pathways regulating synaptic formation and autophagy attenuates Alzheimer’s disease pathology in mice
Source: Front Pharmacol. 2022 Aug 16;13:913971. doi: 10.3389/fphar.2022.913971 (PMC9426773; doi:10.3389/fphar.2022.913971)
Supplement: Supplementary file 9 [file Image4.pdf]

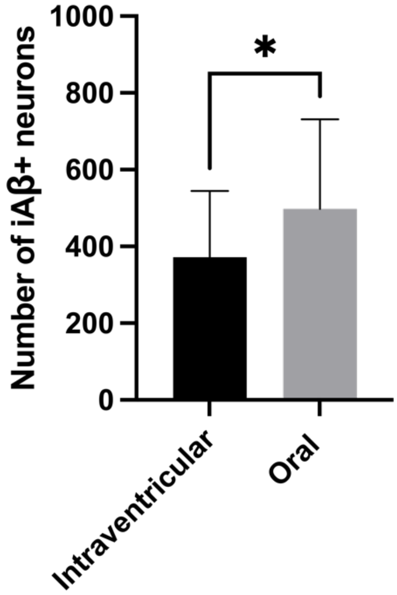

Supplementary Figure 4. The effect of intraventricular and oral administration of Fasudil and Lonafarnib. Mean number of Aβ+ neurons in dSub of 3xTg AD mice after infusions of Lonafarnib and Fasudil via intraventricular microdialysis (*n* = 4) or oral administration (*n* = 3). Intraneuronal Aβ in dSub was quantified from at least 7 brain sections for each animal using Ilastik. Error bars denote ±1 SD, unpaired two-tailed t-test, * $p < 0.05$). Abbreviations; iAβ: intraneuronal amyloid-β.
