# Supplementary material for: Combined targeting of pathways regulating synaptic formation and autophagy attenuates Alzheimer’s disease pathology in mice
Source: Front Pharmacol. 2022 Aug 16;13:913971. doi: 10.3389/fphar.2022.913971 (PMC9426773; doi:10.3389/fphar.2022.913971)
Supplement: Supplementary file 10 [file Image2.pdf]

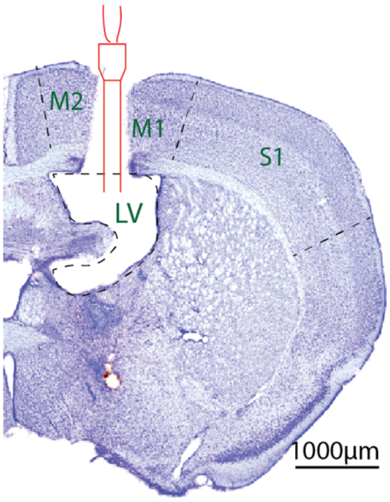

Supplementary Figure 2. Histological verification of probe placement in the lateral ventricle. The microdialysis probe was successfully implanted in LV along the rostrocaudal axis in all animals (*n* = 32). Stereotaxic coordinates: A/P: -0.1mm, M/L: +1.2mm, D/V: -2.75mm. Delineations based on Paxinos & Franklin[1]. Abbreviations; LV: lateral ventricle; M1: primary motor cortex; M2: secondary motor cortex; S1: primary somatosensory cortex.

---

[1] Paxinos, G., and Franklin, K.B. (2004). *The mouse brain in stereotaxic coordinates.* Houston, TX, USA: Gulf Professional Publishing.
