# Supplementary material for: Combined targeting of pathways regulating synaptic formation and autophagy attenuates Alzheimer’s disease pathology in mice
Source: Front Pharmacol. 2022 Aug 16;13:913971. doi: 10.3389/fphar.2022.913971 (PMC9426773; doi:10.3389/fphar.2022.913971)
Supplement: Supplementary file 11 [file Image3.pdf]

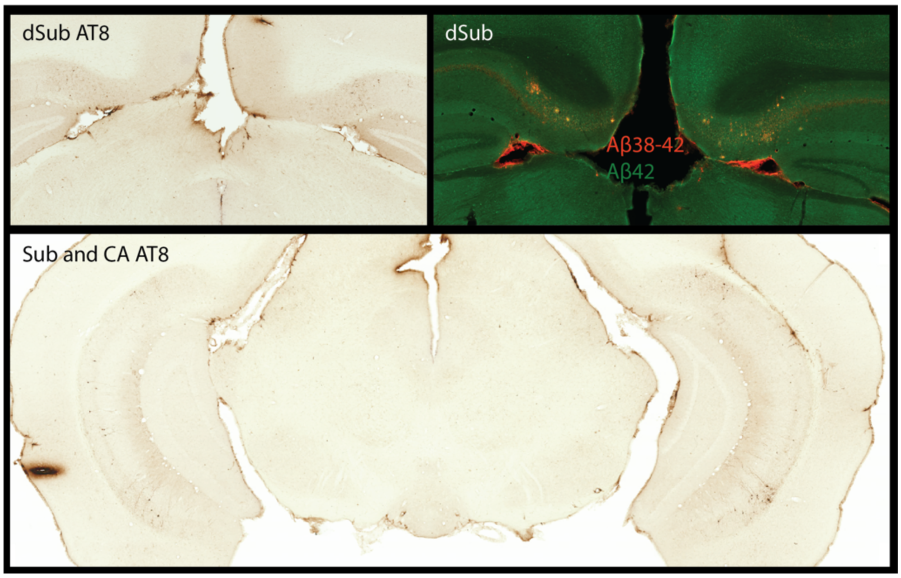

Supplementary Figure 3. No lateralization of neuropathology in 3xTg AD mice. Hyperphosphorylated pathological tau (AT8; brown) in an 18-month-old, and $A\beta_{38\text{-}42}$ (McSA1; red) and $A\beta_{42}$ (IBL $A\beta_{42}$; green) in a 17-month-old 3xTg AD mouse (*n* = 2). Abbreviations; dSub: dorsal subiculum; Sub: subiculum; CA: cornu ammonis.
