# Supplementary material for: Combined targeting of pathways regulating synaptic formation and autophagy attenuates Alzheimer’s disease pathology in mice
Source: Front Pharmacol. 2022 Aug 16;13:913971. doi: 10.3389/fphar.2022.913971 (PMC9426773; doi:10.3389/fphar.2022.913971)
Supplement: Supplementary file 12 [file Table3.pdf]

Supplementary Table 3: Characterization summary for our 3xTg AD mouse colony

Neuropathological marker	Previous characterizations[1,2,3]	Our characterization
**Early intraneuronal Aβ**	Present in CA1, amygdala and neocortex at 4 months-of-age[5,6]	Present in frontal and sensory cortex, hippocampus, and parts of the cerebellum at 1 month-of-age (Supplementary Fig. 8)
**Amyloid plaques**	Present in frontal cortex at 6 months-of-age[5,6]	Present in dorsal subiculum at 13 months-of-age (Supplementary Fig. 8B, C). Presents as dense-core amyloid plaques with surrounding reactive microglia (Supplementary Fig. 9) and MC1 co-labelling (Supplementary Fig. 12)
**Early intraneuronal tau**	Present in hippocampus and amygdala at 1 month-of-age[7]	Present in frontal and sensory cortex, and hippocampus at 1 month-of-age. Early intraneuronal tau decreased with aging (Supplementary Fig. 11A)
**Pre-tangles**	Present in CA1 at 12-15 months-of-age[5,6]	Present in CA1 and caudal subiculum at 13 months-of-age (Supplementary Fig. 11B)
**Neurofibrillary tangles**	Present in CA1 at 18 months-of-age[5,6]	Present in dorsal subiculum, CA1 and entorhinal cortex at 18 months-of-age, and present in entire brain parenchyma by 22 months-of-age (Supplementary Fig. 11C)

[1] Oddo, S., Caccamo, A., Shepherd, J.D., Murphy, M.P., Golde, T.E., Kayed, R., Metherate, R., Mattson, M.P., Akbari, Y., and Laferla, F.M. (2003). Triple-Transgenic Model of Alzheimer's Disease with Plaques and Tangles. *Neuron* 39**,** 409-421.

[2] Billings, L.M., Oddo, S., Green, K.N., Mcgaugh, J.L., and Laferla, F.M. (2005). Intraneuronal Abeta causes the onset of early Alzheimer's disease-related cognitive deficits in transgenic mice. Ibid.45**,** 675-688.

[3] Oh, K.-J., Perez, S.E., Lagalwar, S., Vana, L., Binder, L., and Mufson, E.J. (2010). Staging of Alzheimer's pathology in triple transgenic mice: a light and electron microscopic analysis. *International journal of Alzheimer's disease* 2010**,** 780102.
