# Supplementary material for: Combined targeting of pathways regulating synaptic formation and autophagy attenuates Alzheimer’s disease pathology in mice
Source: Front Pharmacol. 2022 Aug 16;13:913971. doi: 10.3389/fphar.2022.913971 (PMC9426773; doi:10.3389/fphar.2022.913971)
Supplement: Supplementary file 13 [file DataSheet1.pdf]

Supplementary Material

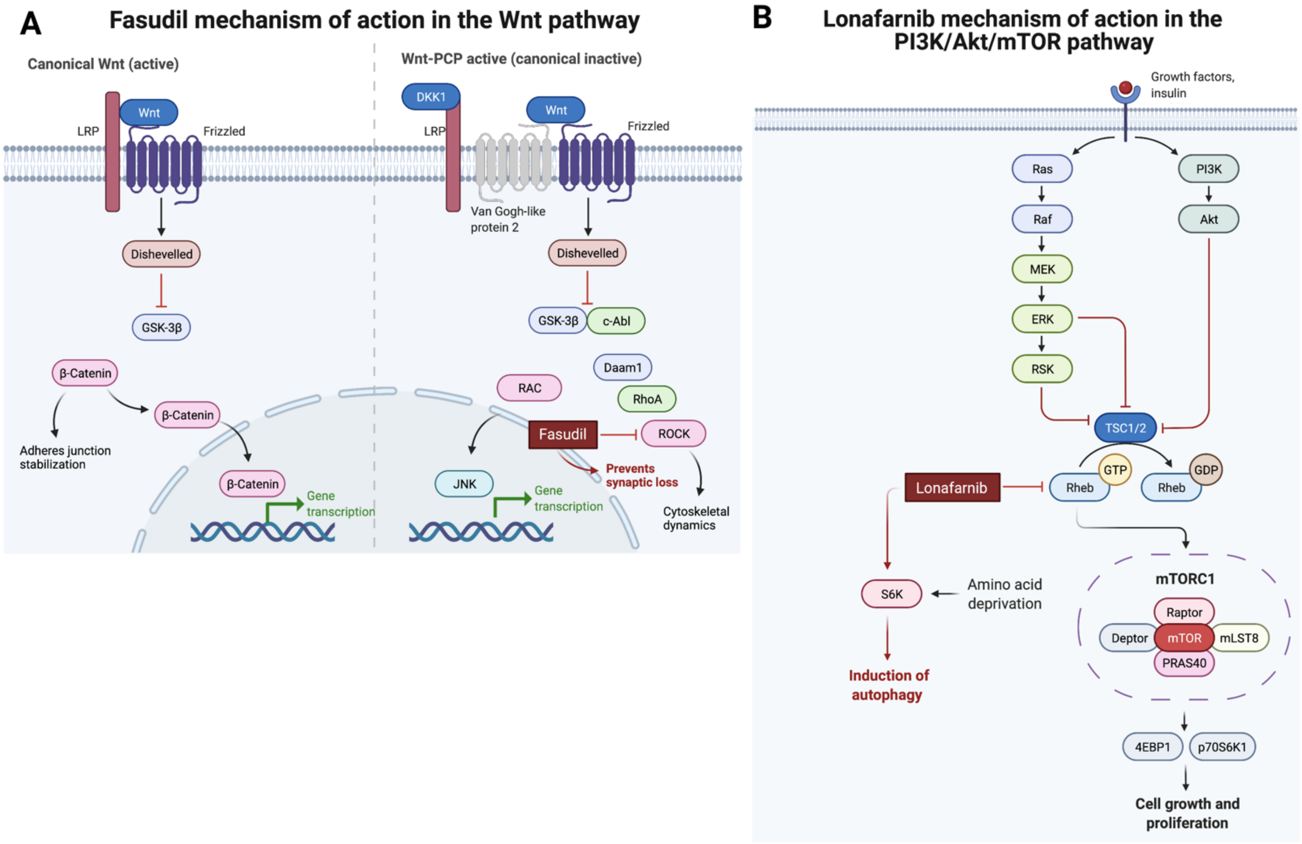

Supplementary Figure 1. Molecular action of Fasudil and Lonafarnib in the Wnt-PCP and PI3K/Akt/mTOR pathway, respectively. (A) Schematic of the canonical Wnt and Wnt-PCP pathways. Aβ has been shown to activate the Wnt-PCP pathway through the ability of Aβ to induce Dkk1. Dkk1 then prevents the binding interaction between LRP6 and frizzled, activating Wnt-PCP signaling and blocking canonical Wnt-β-catenin activity. In the Wnt-PCP pathway, the two arms diverge below disheveled, acting via Daam1/RhoA/ROCK to regulate cytoskeletal dynamics and JNK/c-Jun to regulate gene transcription. Along the same arm of Wnt-PCP signaling acting via Daam1/RhoA/ROCK, binding of a Wnt receptor to frizzled causes disheveled to inhibit the activity of GSK-3β. The ROCK inhibitor Fasudil inhibits the arm of the Wnt-PCP pathway that promotes the retraction of dendritic spines and synapses through Daam1/RhoA/ROCK. Figure adapted from Sellers et al.[1]. Abbreviations; Daam1: disheveled associated activator of morphogenesis 1; Dkk1: Dickkopf-1; GSK-3β: glycogen synthase kinase-3β; JNK: c-Jun N-terminal kinase; LRP: low-density lipoprotein receptor-related protein; PCP: planar cell polarity; RhoA: Ras homolog family member A; ROCK:

---

[1] Sellers, K.J., Elliott, C., Jackson, J., Ghosh, A., Ribe, E., Rojo, A.I., Jarosz-Griffiths, H.H., Watson, I.A., Xia, W., Semenov, M., Morin, P., Hooper, N.M., Porter, R., Preston, J., Al-Shawi, R., Baillie, G., Lovestone, S., Cuadrado, A., Harte, M., Simons, P., Srivastava, D.P., and Killick, R. (2018). Amyloid β synaptotoxicity is Wnt-PCP dependent and blocked by fasudil. *Alzheimers Dement* 14**,** 306-317.

Rho-associated coiled-coil containing protein kinase; Wnt: Wingless-related integration site. **(B) Schematic of the PI3K/Akt/mTOR pathway**. Activation of mTOR results in activation of downstream components (i.e., 4EBP1 and p70S6K1). Lonafarnib works as a farnesyltransferase (farnesylation is a posttranslational modification of proteins) inhibitor which acts as an autophagic inducer by inhibiting mTOR. The mechanisms of action involve Rheb and the PI3K/Akt/mTOR pathway. Rheb acts downstream of TSC1/TSC2 and upstream of mTOR to regulate cell growth and activates S6K during amino acid deprivation via mTOR. Abbreviations; mTOR: mammalian target of rapamycin; Rheb: Ras homologue enriched in brain; PI3K: phosphatidylinositide 3-kinase; Akt: protein kinase B; TSC1/2: tuberous sclerosis complex 1/2; S6K: S6 kinase 1. Figure created with biorender.com.

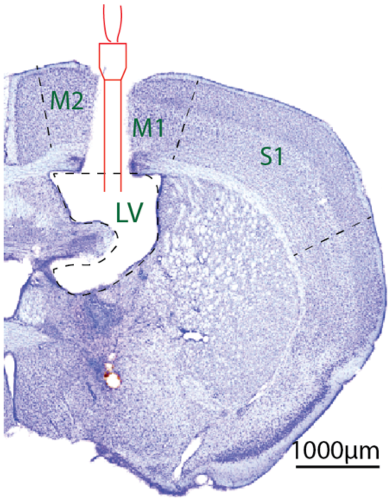

Supplementary Figure 2. Histological verification of probe placement in the lateral ventricle. The microdialysis probe was successfully implanted in LV along the rostrocaudal axis in all animals (*n* = 32). Stereotaxic coordinates: A/P: -0.1mm, M/L: +1.2mm, D/V: -2.75mm. Delineations based on Paxinos & Franklin[2]. Abbreviations; LV: lateral ventricle; M1: primary motor cortex; M2: secondary motor cortex; S1: primary somatosensory cortex.

---

[2] Paxinos, G., and Franklin, K.B. (2004). *The mouse brain in stereotaxic coordinates.* Houston, TX, USA: Gulf Professional Publishing.

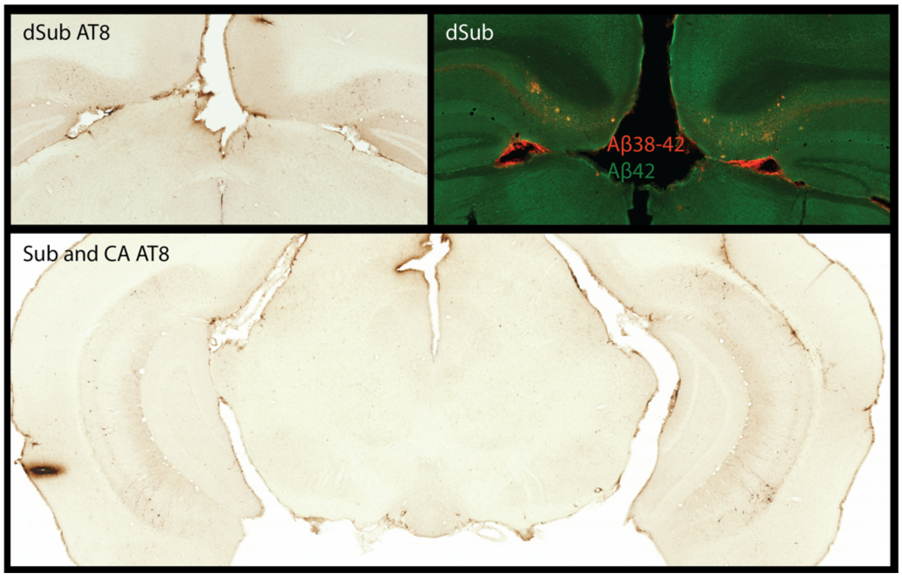

Supplementary Figure 3. No lateralization of neuropathology in 3xTg AD mice. Hyperphosphorylated pathological tau (AT8; brown) in an 18-month-old, and $A\beta_{38-42}$ (McSA1; red) and $A\beta_{42}$ (IBL $A\beta_{42}$; green) in a 17-month-old 3xTg AD mouse ($n = 2$). Abbreviations; dSub: dorsal subiculum; Sub: subiculum; CA: cornu ammonis.

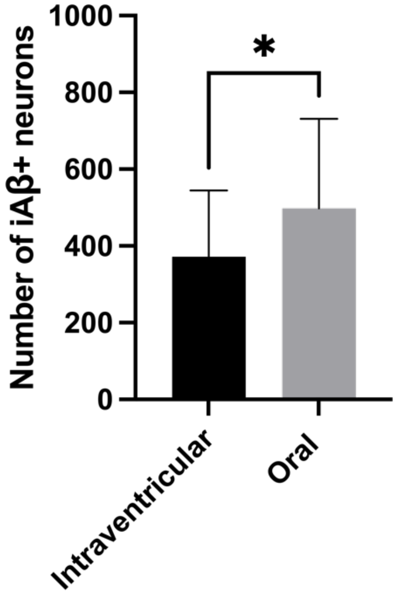

Supplementary Figure 4. The effect of intraventricular and oral administration of Fasudil and Lonafarnib. Mean number of Aβ+ neurons in dSub of 3xTg AD mice after infusions of Lonafarnib and Fasudil via intraventricular microdialysis ($n = 4$) or oral administration ($n = 3$). Intraneuronal Aβ in dSub was quantified from at least 7 brain sections for each animal using Ilastik. Error bars denote ±1 SD, unpaired two-tailed t-test, * $p < 0.05$). Abbreviations; iAβ: intraneuronal amyloid-β.

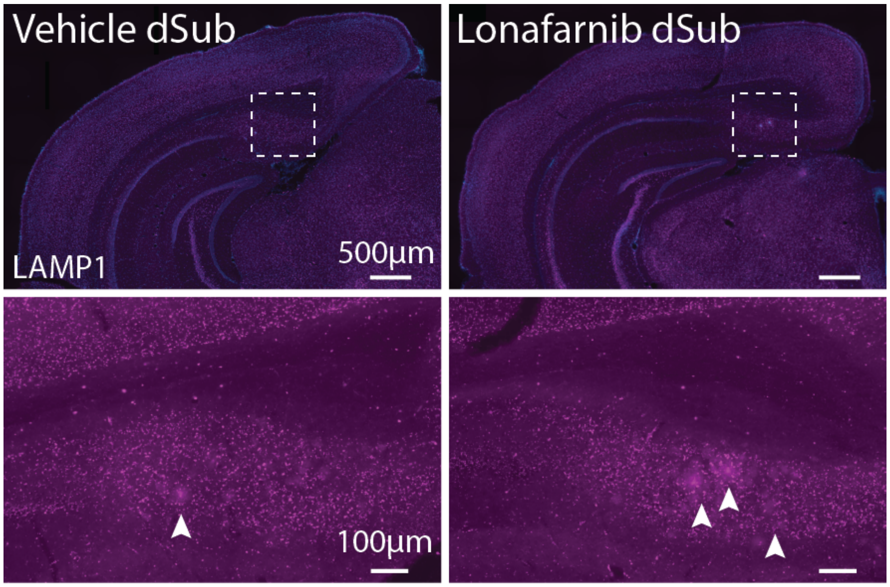

Supplementary Figure 5. LAMP1 (lysosomal marker; purple) immunoreactivity (arrowheads) in dSub in 14-month-old 3xTg AD mice receiving infusions of a vehicle (n = 2) or Lonafarnib (n = 4). Abbreviations; dSub: dorsal subiculum; LAMP1: lysosomal associated membrane protein 1.

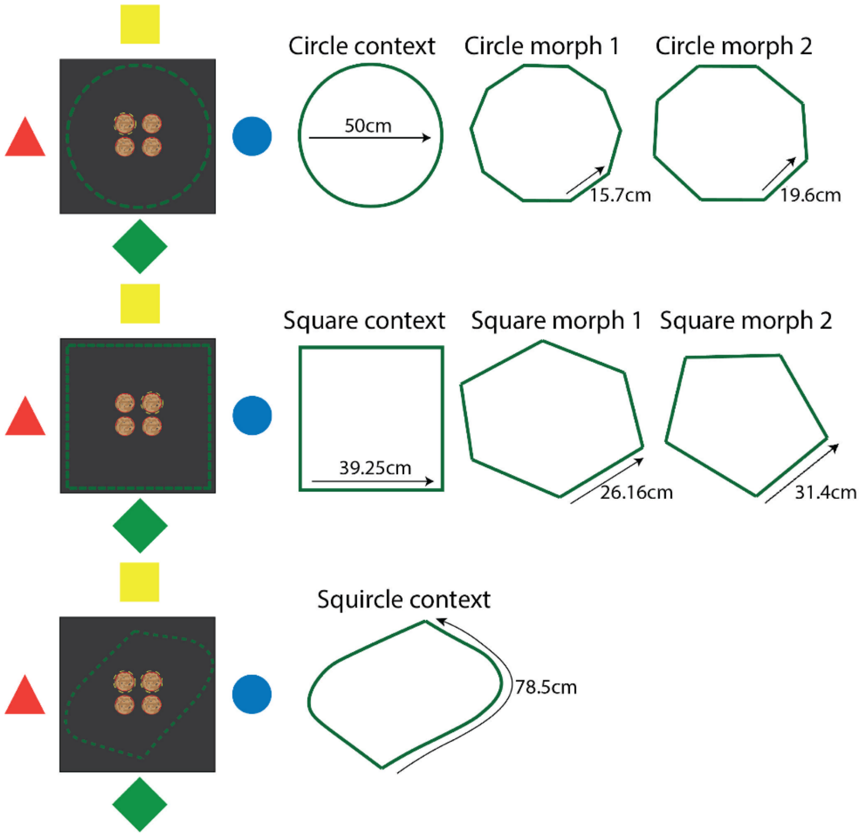

Supplementary Figure 6. Context-dependent spatial memory task design. Mice were initially taught to associate a specific reward location in a square- and a circle-chamber. The reward was buried in one of four cups with ginger-scented bedding. A trial was assessed as correct if the mice dug in the

reward location associated with each chamber. If they passed the training phase (66.6 % correct digging), their contextual memory performance was tested in morph-chambers: a decagon (circle morph 1), an octagon (circle morph 2), a hexagon (square morph 1), and a pentagon (square morph 2). Mice were tested in morph-chambers for 4 days, with 8 sessions a day. If they were able to complete the morph testing, they were tested in a Squircle-chamber on the fifth day. Since the Squircle equally resembles the square- and a circle-chambers, a trial was considered correct if the mouse dug either of the reward locations attributable to the square- or circle-chamber. Figure adapted by permission from Nora Cecilie Ebbesen.

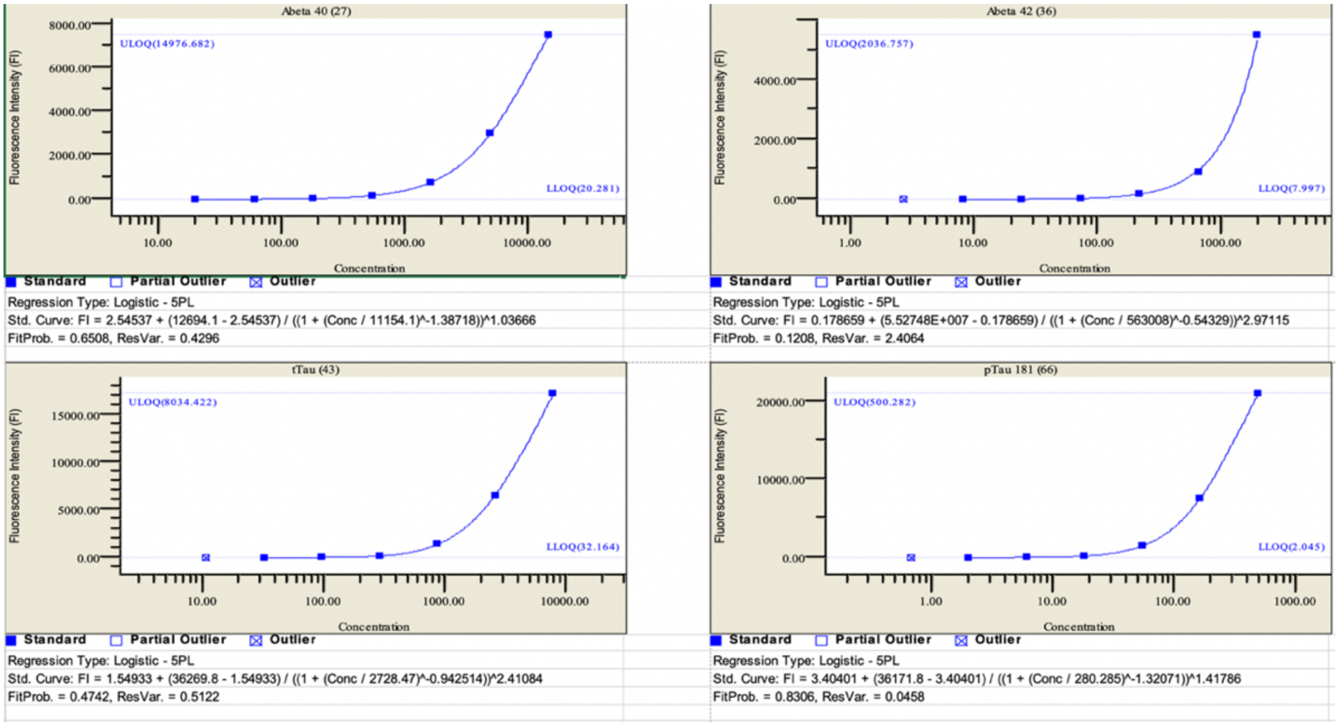

Supplementary Figure 7. LLOQ levels of CSF proteins. Lower limit of quantification (LLOQ) for Aβ_{40}, Aβ_{42}, t-tau, and p-tau from standard curves. Abbreviations; Aβ: amyloid-β; t-tau: total tau; p-tau: phosphorylated tau.

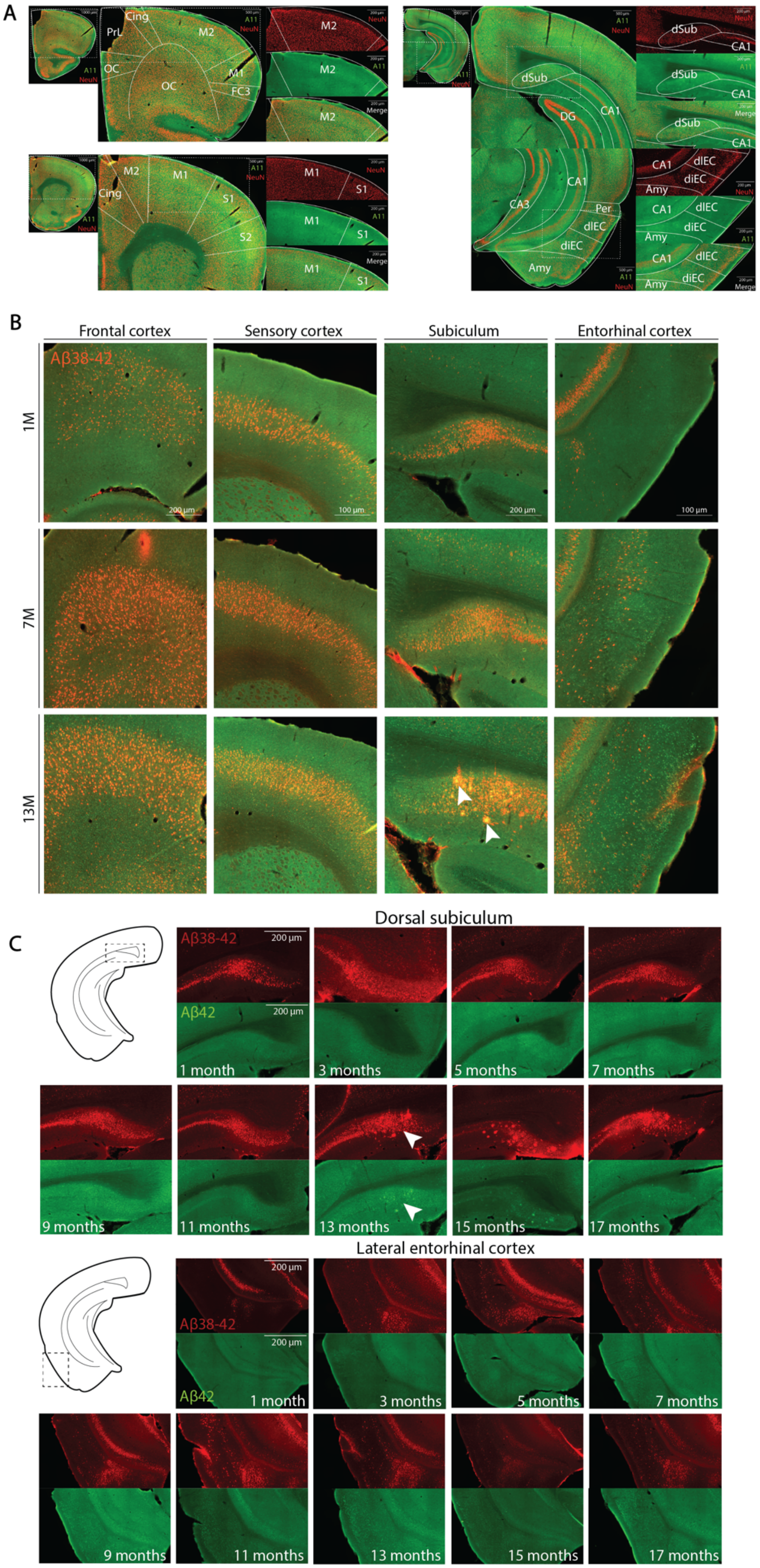
A
Cing
PrL
M2
OC
M1
FC3
A11
NeuN
Merge
S1
S2
dSub
CA1
DG
CA3
Per
dlEC
diEC
Amy
B
Frontal cortex
Sensory cortex
Subiculum
Entorhinal cortex
Aβ38-42
1M
7M
13M
200 µm
100 µm
C
Dorsal subiculum
Aβ42
1 month
3 months
5 months
7 months
9 months
11 months
13 months
15 months
17 months
Lateral entorhinal cortex

Supplementary Figure 8. Characterization of Aβ in the brain of our 3xTg AD mouse colony. (A) A11 (oligomeric Aβ specific; green) and NeuN (nuclei specific; red) immunoreactivity in the 3xTg AD mouse model at 1-month-of-age. At this age, there is little-to-none A11 immunoreactivity in frontal and sensory areas of the brain (left), as well as in the hippocampal and parahippocampal region (right). **(B)** $A\beta_{38\text{-}42}$ immunoreactivity in frontal cortex, sensory cortex, Sub, and EC in the 3xTg AD mouse model at 1, 7 and 13 months of age. $A\beta_{38\text{-}42}$ (McSA1; red) and $A\beta_{42}$ (IBL $A\beta_{42}$; green) immunoreactivity in the 3xTg AD mouse model. Amyloid plaques immunoreactive to $A\beta_{38\text{-}42}$ (McSA1 antibody) are first apparent at 13 months of age in subiculum. **(C)** $A\beta_{38\text{-}42}$ immunoreactivity in the 3xTg AD mouse model at various ages in the dSub and LEC. $A\beta_{38\text{-}42}$ (McSA1; red) and $A\beta_{42}$ (IBL $A\beta_{42}$; green) immunoreactivity in the 3xTg AD mouse model. According to the ABC scoring system, diffuse amyloid plaques is scored in the cerebral cortex, hippocampus, striatum, midbrain, brainstem, and cerebellum according to protocols established by Thal et al.[3] resulting in a Thal phase 0-5, which is translated into the NIA-AA score of A0-A3. Amyloid plaques immunoreactive to $A\beta_{38\text{-}42}$ (McSA1 antibody) are first apparent at 13 months of age in dSub, whereas plaques immunoreactive to $A\beta_{42}$ (IBL $A\beta_{42}$ antibody) are first apparent at 15 months of age in dSub. Abbreviations; S1: primary somatosensory cortex; S2: secondary somatosensory cortex; Olf: olfactory area; OC: orbital cortex; PrL: prelimbic cortex; Cing: cingulate cortex; M1: primary motor cortex; M2: secondary motor cortex; FC3: frontal cortex area 3; Ins: insular cortex; CA1-3: cornu ammonis field 1-3; Amy: amygdala; diEC: dorsal intermediate entorhinal cortex; dlEC: dorsolateral entorhinal cortex; dSub: dorsal subiculum; PER: perirhinal cortex. Aβ: amyloid-β; Sub: subiculum; LEC: lateral entorhinal cortex.

[3] Thal, D.R., Rub, U., Orantes, M., and Braak, H. (2002). Phases of A beta-deposition in the human brain and its relevance for the development of AD. *Neurology* 58**,** 1791-1800.

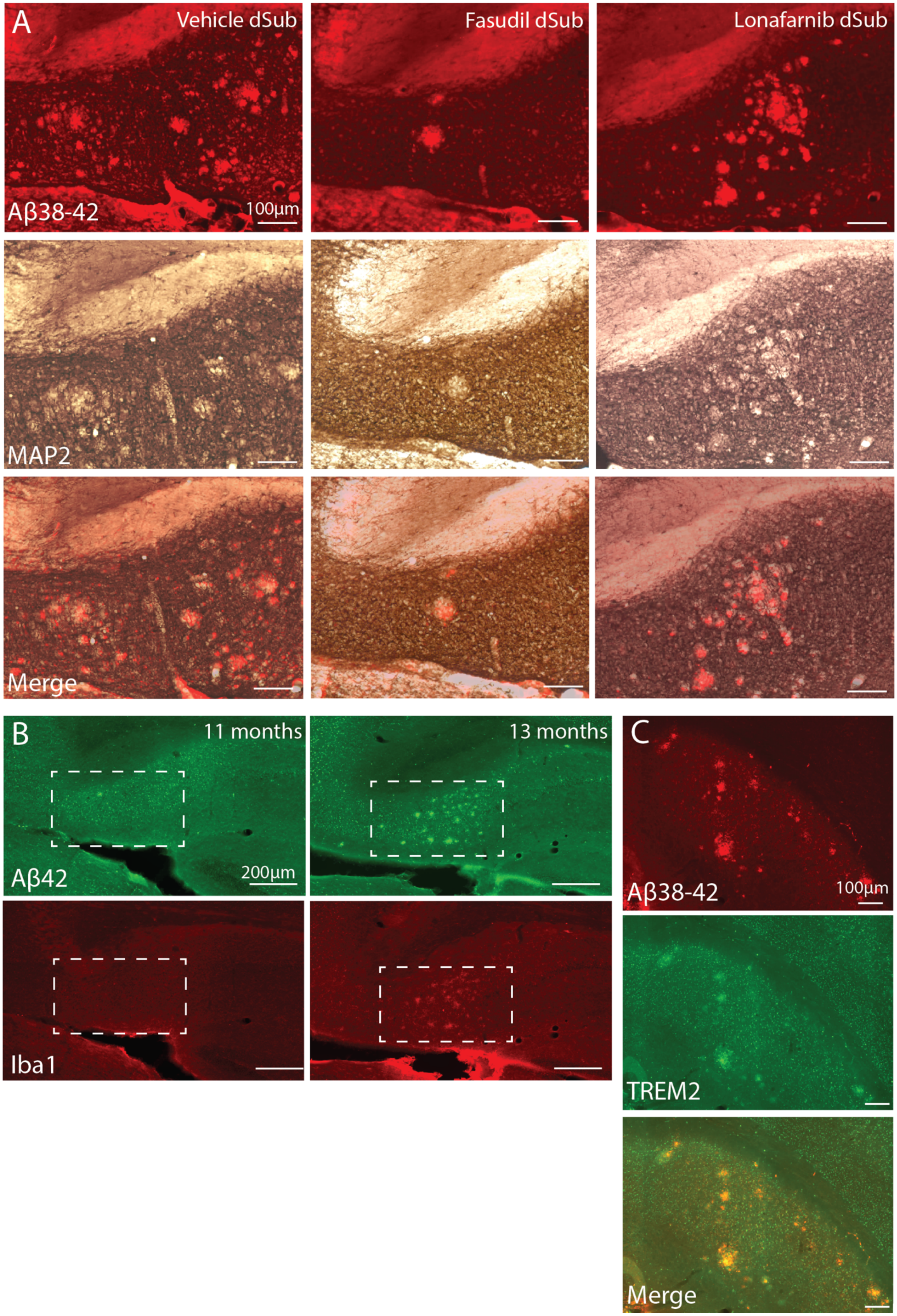
A
Vehicle dSub
Fasudil dSub
Lonafarnib dSub
Aβ38-42
100µm
MAP2
Merge
B
11 months
13 months
Aβ42
200µm
Iba1
C
Aβ38-42
100µm
TREM2
Merge

Supplementary Figure 9. Dense-core amyloid plaques after Fasudil and Lonafarnib infusions, and depiction of associated microglial activation surrounding dense-core amyloid plaques. (A) A$\beta_{38\text{-}42}$ (red; McSA1) immunolabelling for amyloid plaques (top panel) and DAB staining (brown) for microtubule-associated protein 2 middle panel; MAP2). McSA1 and the absence of MAP2 staining colocalized (bottom panel), confirming previous research indicating that MAP2 processes are absent within dense-core amyloid plaques. **(B)** Aβ_{42} (IBL Aβ_{42}; green) and Iba1 (microglial marker; red) immunoreactivity in dSub at 11- and 13-months-of-age in the 3xTg AD mouse model. **(C)** A$\beta_{38\text{-}42}$ (McSA1; red) and TREM2 (microglial receptor; green) immunoreactivity in dSub at 13-months-of-age. Abbreviations; Aβ: amyloid-β; dSub: dorsal subiculum; Iba1: Ionized calcium-binding adapter molecule 1; TREM2: triggering receptor expressed on myeloid cells 2.

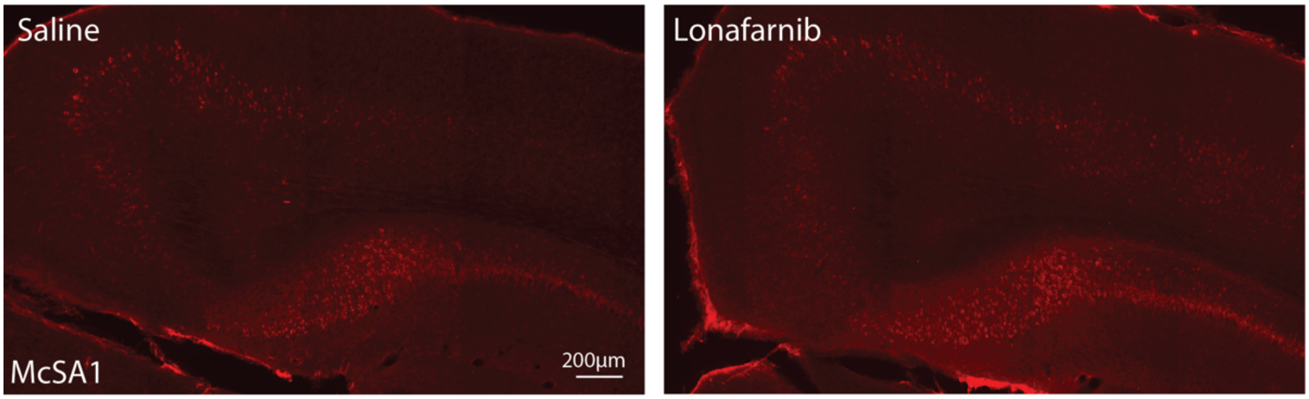

Supplementary Figure 10. Intraneuronal Aβ in dSub after Lonafarnib infusions. Lonafarnib did not affect the number of A$\beta_{38\text{-}42}$+ neurons (McSA1; red) in dSub in 6-month-old 3xTg AD mice (*n* = 2).

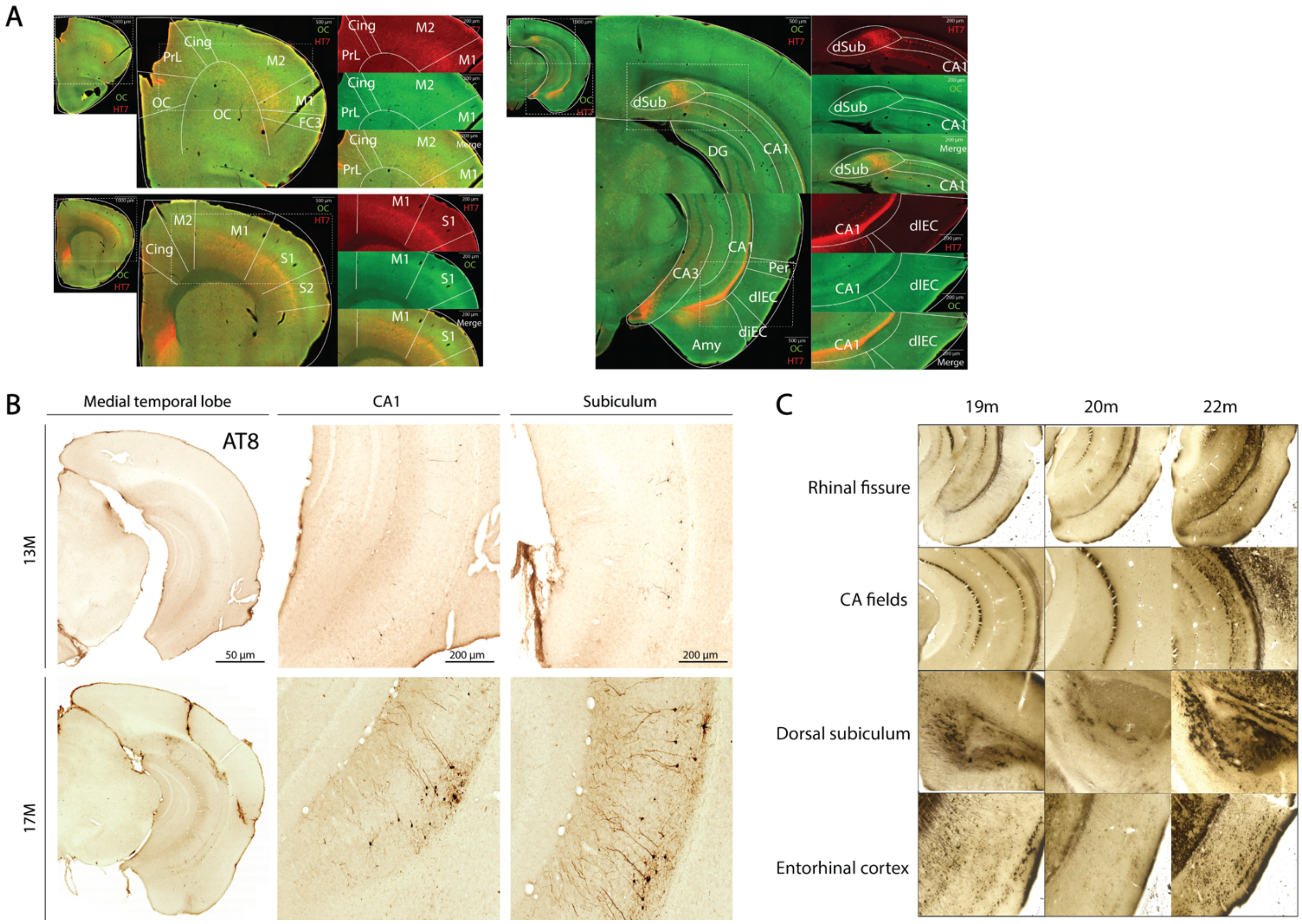

Supplementary Figure 11. Characterization of tau in the brain of our 3xTg AD mouse colony. (A) MAPT (HT7; red) and fibrillar Aβ (OC; green) immunoreactivity in the entire brain at 1-month-of-age in 3xTg AD mice. **(B)** AT8 (detects phosphorylated tau proteins at serine 202 and threonine 205 residues; DAB) immunoreactivity in the 3xTg AD mouse at 13- and 17-months-of-age. An ABC score for NFTs is determined in the trans-entorhinal area, CA, fronto-parietal cortex, and primary visual cortex to generate a Braak stage[4], which is translated into the NIA-AA score of B0-B3. Most existing mouse models do not generate NFTs, so the National Institutes of Health (NIH) have developed a modified B score for p-tau pathology, including distribution of cytoplasmic neuronal tau such as pre-tangles and threads. **(C)** Gallyas-silver staining in the 3xTg AD mouse at 19-, 20- and 22-months-of-age. Abbreviations; S1: primary somatosensory cortex; S2: secondary somatosensory cortex; Olf: olfactory area; OC: orbital cortex; PrL: prelimbic cortex; Cing: cingulate cortex; M1: primary motor cortex; M2: secondary motor cortex; FC3: frontal cortex area 3; Ins: insular cortex; CA1: cornu ammonis field 1; CA2: cornu ammonis field 2; CA3: cornu ammonis field 3; Amy: amygdala; diEC: dorsal intermediate entorhinal cortex; dlEC: dorsolateral entorhinal cortex; dSub: dorsal subiculum; PER: perirhinal cortex; M: months.

---

[4] Braak, H., and Braak, E. (1991). Neuropathological stageing of Alzheimer-related changes. *Acta Neuropathol* 82**,** 239-259.

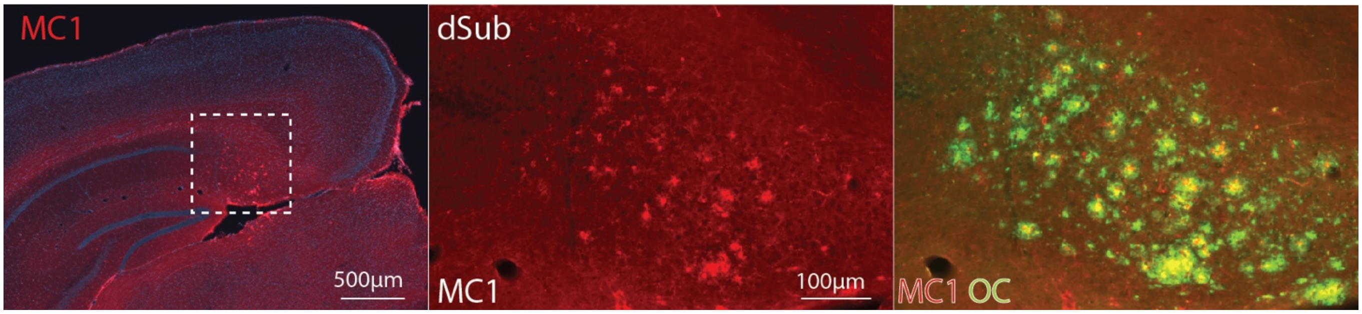

Supplementary Figure 12. MC1+ amyloid plaques in 3xTg AD mice. Conformation-specific tau (MC1; red) and fibrillar Aβ (OC; green) immunoreactivity in dSub at 13-months-of-age. Abbreviations; dSub: dorsal subiculum.

Supplementary Table 1: Key resources

Reagent type (species) or resource	Designation	Information	Identifiers/reference
**Strain, strain background (Mus musculus)**	3xTg AD	B6;129-Psen1^{tm1Mpm}Tg(APPSwe,tauP301L)1Lfa/Mmjax	MMRRC Strain #034830-JAX; RRID: MMRRC_034830-MU; PMID: 12895417
**Strain, strain background (Mus musculus)**	B6129	B6129SF2/J	Strain #:101045; RRID:IMSR_JAX:101045
**Genetic reagent (virus)**	AAV-CBA-GFP-2A-P301L-Tau (serotype 8)	Viral Vector Core at Kavli Institute for Systems Neuroscience; contact Dr. Raveendran, rajeevkumar.r.nair@ntnu.no	Gifted by Bradley Hyman's lab, Harvard Medical School; PMID: 31249873
**Antibody**	Mouse anti-Aβ (McSA1)*	Targets the N-terminal amino acids 1-12 of human Aβ	MediMabs Cat# MM-0015-1P, RRID:AB_1807985
**Antibody**	Anti-$A\beta_{42}$ (rabbit polyclonal)*	$A\beta_{42}$ (pre-oligomers)	Tecan (IBL) Cat# JP28051, RRID:AB_2341462
**Antibody**	Anti-oligomer A11 (rabbit polyclonal)	Soluble $A\beta_{40}$/oligomeric $A\beta_{42}$ (prefibrils)	Thermo Fisher Scientific Cat# AHB0052, RRID:AB_2536236
**Antibody**	Anti-amyloid fibrils OC (rabbit polyclonal)*	Amyloid fibrils/fibrillar oligomers (protofibrils)	Millipore Cat# AB2286, RRID:AB_1977024
**Antibody**	Anti-Iba1 (mouse monoclonal)	Ionized calcium binding adaptor molecule 1 (Iba1)	Abcam Cat# ab15690, RRID:AB_2224403
**Antibody**	Anti-TREM2 (rabbit monoclonal)	TREM2 receptor	Thermo Fisher Scientific Cat# MA5-30971, RRID:AB_2786636

**Antibody**	Anti-MAP2 (rabbit monoclonal)	Microtubule-associated protein 2	Abcam Cat# ab183830, RRID:AB_2895301; PMID: 12083391
**Antibody**	Anti-LAMP1 (rabbit polyclonal)	Lysosomal associated membrane protein 1	Sigma-Aldrich Cat# L1418, RRID:AB_477157
**Antibody**	Anti-phospho-tau AT8 (mouse monoclonal)	Tau phosphorylated at serine 202 and threonine 205	Thermo Fisher Scientific Cat# MN1020, RRID:AB_223647
**Antibody**	Anti-tau HT7 (mouse monoclonal)	Recognized $tau_{159\text{-}163}$ and does not cross-react with murine tau	Thermo Fisher Scientific Cat# MN1000, RRID:AB_2314654; PMID: 1729400
**Antibody**	Anti-tau MC1 (mouse monoclonal)	Conformation specific, detects misfolded tau relevant to tauopathy	Gifted by Peter Davies, Department of Pathology, Albert Einstein College of Medicine
**Antibody**	Anti-NeuN (rabbit monoclonal)	Neuronal labelling	Abcam Cat# ab177487, RRID:AB_2532109
**Antibody**	Goat anti-mouse IgG (AF 657)	Secondary antibody	Thermo Fisher Scientific Cat# A-21235, RRID:AB_2535804
**Antibody**	Goat anti-mouse IgG (AF 546)	Secondary antibody	Thermo Fisher Scientific Cat# A-11030, RRID:AB_2534089
**Antibody**	Goat anti-mouse IgG (AF 488)	Secondary antibody	Thermo Fisher Scientific Cat# A28175, RRID:AB_2536161
**Antibody**	Goat anti-rabbit IgG (AF 488)	Secondary antibody	Molecular Probes Cat# A-11008, RRID:AB_143165
**Antibody**	Goat anti-rabbit IgG (AF 546)	Secondary antibody	Thermo Fisher Scientific Cat# A-11035, RRID:AB_2534093
**Chemical compound, drug**	Fasudil	Rho kinase inhibitor	Selleck Chemicals Cat# S1573; PMID: 29055813
**Chemical compound, drug**	Lonafarnib	Farnesyltransferase inhibitor with antitumor activity	Cayman Chemical Cat# CAY11746-1 mg; PMID: 30918111
**Chemical compound, drug**	DAPI (4',6-Diamidino-2-Phenylindole, Dihydrochloride)	Nuclear and chromosome counterstain	Thermo Fisher Scientific Cat# D1306, RRID:AB_2629482

**Chemical compound, drug**	Nissl (cresyl violet)	RNA labelling	Bjorkli 2019 doi: https://protocols.io/view/ihc-ad-neuropathology-protocol-btbmnik6.
**Chemical compound, drug**	Gallyas-silver staining	Modified silver impregnation of NFTs	Bjorkli & Lagartos-Donate 2022 doi: dx.doi.org/10.17504/protocols.io.b44cqysw.
**Chemical compound, drug**	DAB	Chromogen for detecting antibodies	Bjorkli 2022 doi: dx.doi.org/10.17504/protocols.io.b44fqytn.
**Software**	GraphPad Prism, version 9	Statistics and data visualization software	
**Software**	Zeiss ZEN lite	Microscope software	
**Software**	Ilastik	Cell counting software	PMID: 31570887
**Software**	ANY-maze – Stoelting Co.	Video tracking software	

* Labels amyloid plaques

Supplementary Table 2: Sample size for experimental conditions

Experimental condition	4 months-old	6 months-old	14 months-old
**Fasudil treatment (n)**		6	4
**Lonafarnib treatment (n)**		4*	2
**Combinatorial treatment (n)**	6^{+}		
**Saline (vehicle) treatment (n)**	5^{+}	6*	2

* 2 of these animals were injected with AAV-tau
$^{+}$ 3-4 of these animals underwent behavioral testing

Supplementary Table 3: Characterization summary for our 3xTg AD mouse colony

Neuropathological marker	Previous characterizations[5,6,7]	Our characterization
**Early intraneuronal Aβ**	Present in CA1, amygdala and neocortex at 4 months-of-age[5,6]	Present in frontal and sensory cortex, hippocampus, and parts of the cerebellum at 1 month-of-age (Supplementary Fig. 8)
**Amyloid plaques**	Present in frontal cortex at 6 months-of-age[5,6]	Present in dorsal subiculum at 13 months-of-age (Supplementary Fig. 8B, C). Presents as dense-core amyloid plaques with surrounding reactive microglia (Supplementary Fig. 9) and MC1 co-labelling (Supplementary Fig. 12)
**Early intraneuronal tau**	Present in hippocampus and amygdala at 1 month-of-age[7]	Present in frontal and sensory cortex, and hippocampus at 1 month-of-age. Early intraneuronal tau decreased with aging (Supplementary Fig. 11A)
**Pre-tangles**	Present in CA1 at 12-15 months-of-age[5,6]	Present in CA1 and caudal subiculum at 13 months-of-age (Supplementary Fig. 11B)
**Neurofibrillary tangles**	Present in CA1 at 18 months-of-age[5,6]	Present in dorsal subiculum, CA1 and entorhinal cortex at 18 months-of-age, and present in entire brain parenchyma by 22 months-of-age (Supplementary Fig. 11C)

[5] Oddo, S., Caccamo, A., Shepherd, J.D., Murphy, M.P., Golde, T.E., Kayed, R., Metherate, R., Mattson, M.P., Akbari, Y., and Laferla, F.M. (2003). Triple-Transgenic Model of Alzheimer's Disease with Plaques and Tangles. *Neuron* 39**,** 409-421.

[6] Billings, L.M., Oddo, S., Green, K.N., Mcgaugh, J.L., and Laferla, F.M. (2005). Intraneuronal Abeta causes the onset of early Alzheimer's disease-related cognitive deficits in transgenic mice. Ibid.45**,** 675-688.

[7] Oh, K.-J., Perez, S.E., Lagalwar, S., Vana, L., Binder, L., and Mufson, E.J. (2010). Staging of Alzheimer's pathology in triple transgenic mice: a light and electron microscopic analysis. *International journal of Alzheimer's disease* 2010**,** 780102.
