# Supplementary material for: Combined targeting of pathways regulating synaptic formation and autophagy attenuates Alzheimer’s disease pathology in mice
Source: Front Pharmacol. 2022 Aug 16;13:913971. doi: 10.3389/fphar.2022.913971 (PMC9426773; doi:10.3389/fphar.2022.913971)
Supplement: Supplementary file 15 [file Image7.pdf]

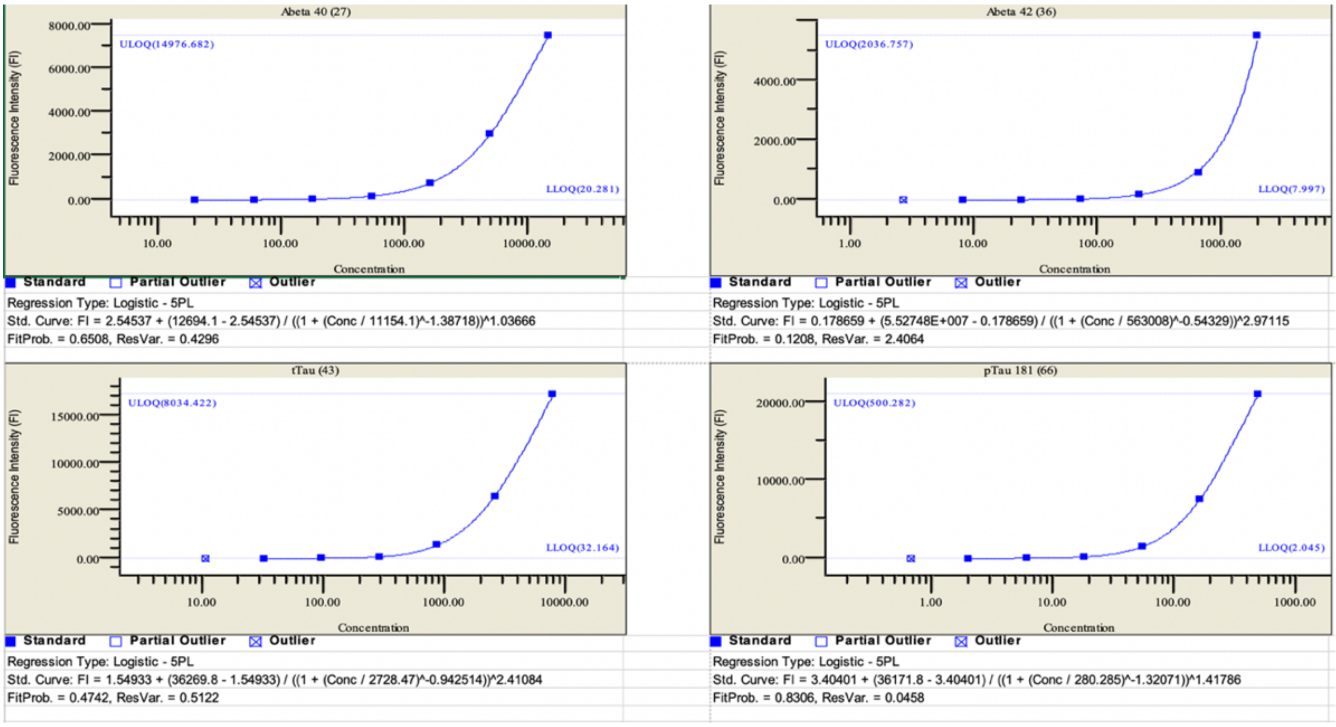

Supplementary Figure 7. LLOQ levels of CSF proteins. Lower limit of quantification (LLOQ) for Aβ_{40}, Aβ_{42}, t-tau, and p-tau from standard curves. Abbreviations; Aβ: amyloid-β; t-tau: total tau; p-tau: phosphorylated tau.
