# Supplementary material for: Combined targeting of pathways regulating synaptic formation and autophagy attenuates Alzheimer’s disease pathology in mice
Source: Front Pharmacol. 2022 Aug 16;13:913971. doi: 10.3389/fphar.2022.913971 (PMC9426773; doi:10.3389/fphar.2022.913971)
Supplement: Supplementary file 16 [file Image1.pdf]

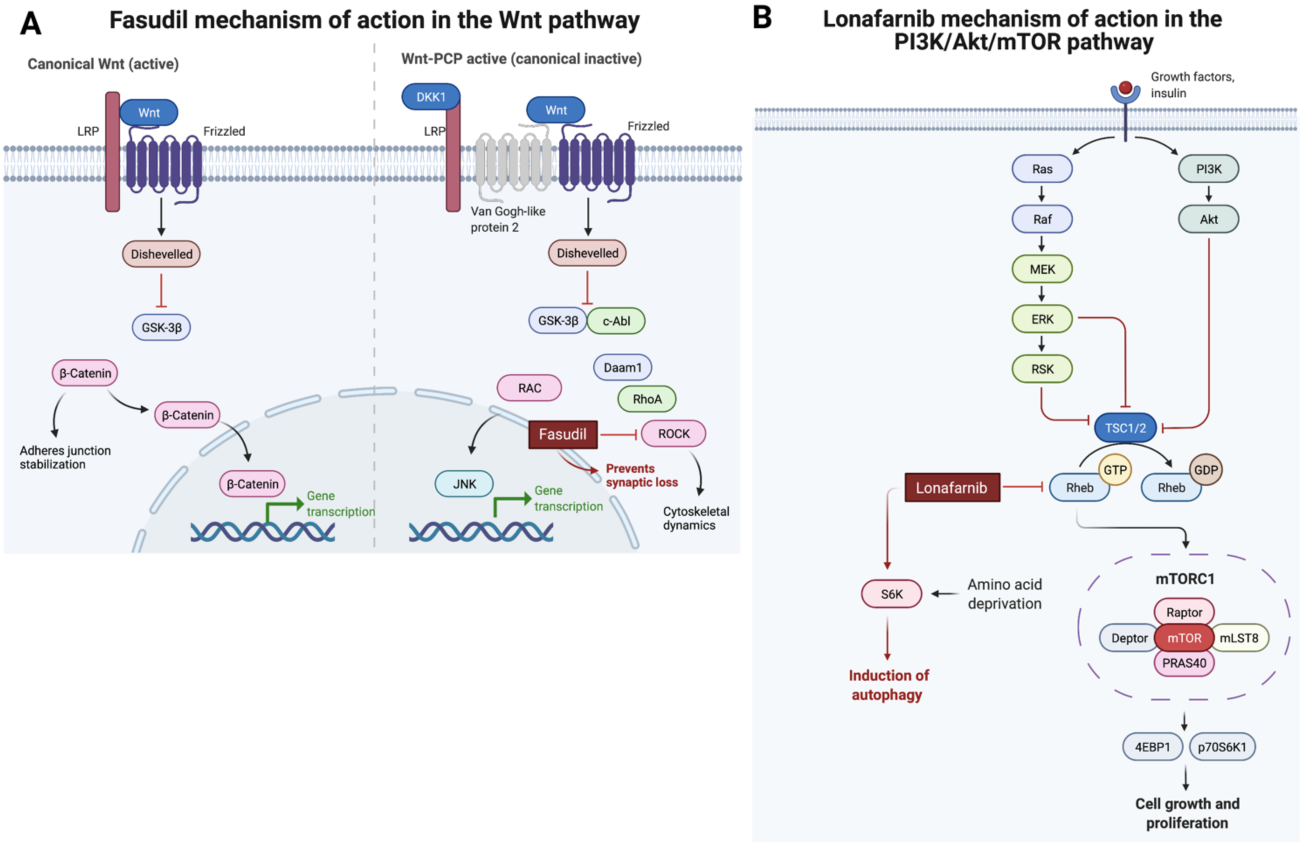

Supplementary Figure 1. Molecular action of Fasudil and Lonafarnib in the Wnt-PCP and PI3K/Akt/mTOR pathway, respectively. (A) Schematic of the canonical Wnt and Wnt-PCP pathways. Aβ has been shown to activate the Wnt-PCP pathway through the ability of Aβ to induce Dkk1. Dkk1 then prevents the binding interaction between LRP6 and frizzled, activating Wnt-PCP signaling and blocking canonical Wnt-β-catenin activity. In the Wnt-PCP pathway, the two arms diverge below disheveled, acting via Daam1/RhoA/ROCK to regulate cytoskeletal dynamics and JNK/c-Jun to regulate gene transcription. Along the same arm of Wnt-PCP signaling acting via Daam1/RhoA/ROCK, binding of a Wnt receptor to frizzled causes disheveled to inhibit the activity of GSK-3β. The ROCK inhibitor Fasudil inhibits the arm of the Wnt-PCP pathway that promotes the retraction of dendritic spines and synapses through Daam1/RhoA/ROCK. Figure adapted from Sellers et al.[1]. Abbreviations; Daam1: disheveled associated activator of morphogenesis 1; Dkk1: Dickkopf-1; GSK-3β: glycogen synthase kinase-3β; JNK: c-Jun N-terminal kinase; LRP: low-density lipoprotein receptor-related protein; PCP: planar cell polarity; RhoA: Ras homolog family member A; ROCK: Rho-associated coiled-coil containing protein kinase; Wnt: Wingless-related integration site. **(B) Schematic of the PI3K/Akt/mTOR pathway**. Activation of mTOR results in activation of downstream components (i.e., 4EBP1 and p70S6K1). Lonafarnib works as a farnesyltransferase (farnesylation is a posttranslational modification of proteins) inhibitor which acts as an autophagic inducer by inhibiting mTOR. The mechanisms of action involve Rheb and the PI3K/Akt/mTOR pathway. Rheb acts downstream of TSC1/TSC2 and upstream

---

[1] Sellers, K.J., Elliott, C., Jackson, J., Ghosh, A., Ribe, E., Rojo, A.I., Jarosz-Griffiths, H.H., Watson, I.A., Xia, W., Semenov, M., Morin, P., Hooper, N.M., Porter, R., Preston, J., Al-Shawi, R., Baillie, G., Lovestone, S., Cuadrado, A., Harte, M., Simons, P., Srivastava, D.P., and Killick, R. (2018). Amyloid β synaptotoxicity is Wnt-PCP dependent and blocked by fasudil. *Alzheimers Dement* 14**,** 306-317.

of mTOR to regulate cell growth and activates S6K during amino acid deprivation via mTOR. Abbreviations; mTOR: mammalian target of rapamycin; Rheb: Ras homologue enriched in brain; PI3K: phosphatidylinositide 3-kinase; Akt: protein kinase B; TSC1/2: tuberous sclerosis complex 1/2; S6K: S6 kinase 1. Figure created with biorender.com.
