# Supplementary material for: Combined targeting of pathways regulating synaptic formation and autophagy attenuates Alzheimer’s disease pathology in mice
Source: Front Pharmacol. 2022 Aug 16;13:913971. doi: 10.3389/fphar.2022.913971 (PMC9426773; doi:10.3389/fphar.2022.913971)
Supplement: Supplementary file 14 [file Image11.pdf]

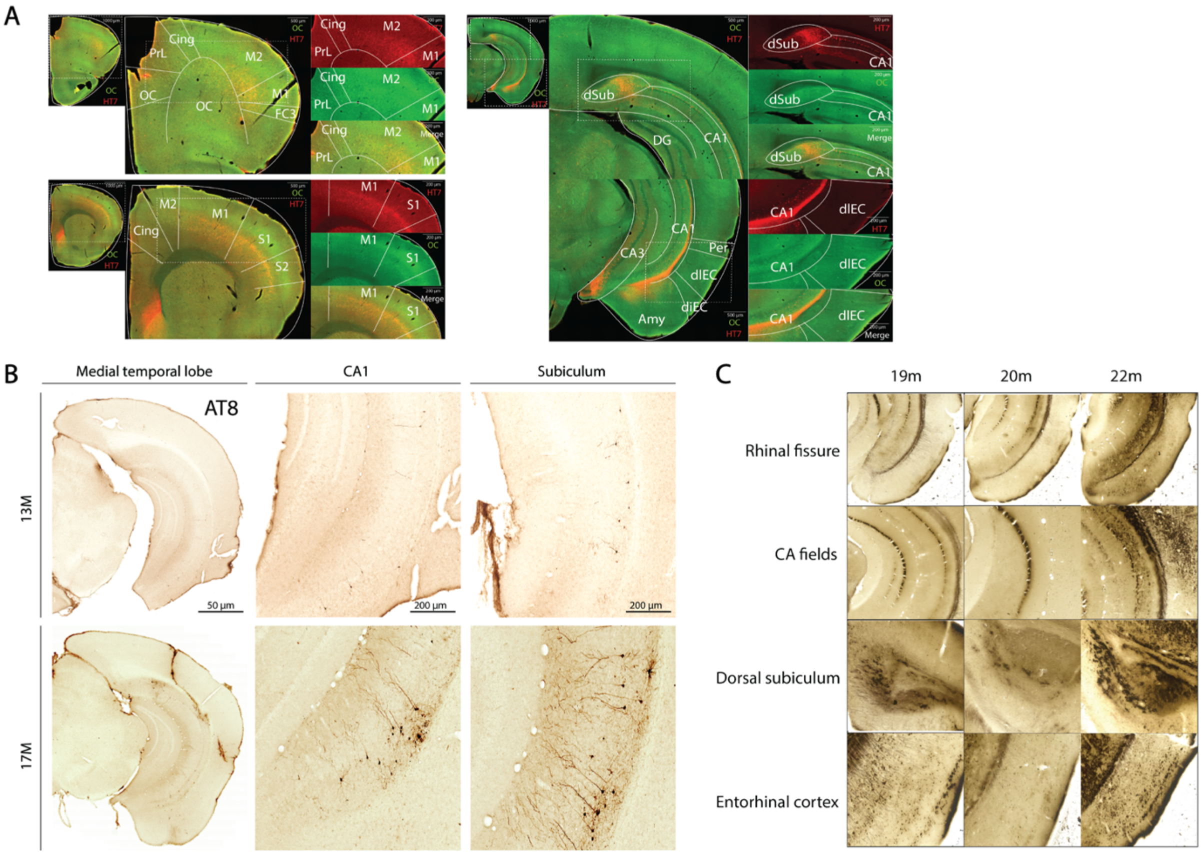

Supplementary Figure 11. Characterization of tau in the brain of our 3xTg AD mouse colony. (A) MAPT (HT7; red) and fibrillar Aβ (OC; green) immunoreactivity in the entire brain at 1-month-of-age in 3xTg AD mice. **(B)** AT8 (detects phosphorylated tau proteins at serine 202 and threonine 205 residues; DAB) immunoreactivity in the 3xTg AD mouse at 13- and 17-months-of-age. An ABC score for NFTs is determined in the trans-entorhinal area, CA, fronto-parietal cortex, and primary visual cortex to generate a Braak stage[1], which is translated into the NIA-AA score of B0-B3. Most existing mouse models do not generate NFTs, so the National Institutes of Health (NIH) have developed a modified B score for p-tau pathology, including distribution of cytoplasmic neuronal tau such as pre-tangles and threads. **(C)** Gallyas-silver staining in the 3xTg AD mouse at 19-, 20- and 22-months-of-age. Abbreviations; S1: primary somatosensory cortex; S2: secondary somatosensory cortex; Olf: olfactory area; OC: orbital cortex; PrL: prelimbic cortex; Cing: cingulate cortex; M1: primary motor cortex; M2: secondary motor cortex; FC3: frontal cortex area 3; Ins: insular cortex; CA1: cornu ammonis field 1; CA2: cornu ammonis field 2; CA3: cornu ammonis field 3; Amy: amygdala; diEC: dorsal intermediate entorhinal cortex; dlEC: dorsolateral entorhinal cortex; dSub: dorsal subiculum; PER: perirhinal cortex; M: months.

[1] Braak, H., and Braak, E. (1991). Neuropathological stageing of Alzheimer-related changes. *Acta Neuropathol* 82**,** 239-259.
